# A Resource Allocation Trade-Off between Virulence and Proliferation Drives Metabolic Versatility in the Plant Pathogen *Ralstonia solanacearum*

**DOI:** 10.1371/journal.ppat.1005939

**Published:** 2016-10-12

**Authors:** Rémi Peyraud, Ludovic Cottret, Lucas Marmiesse, Jérôme Gouzy, Stéphane Genin

**Affiliations:** LIPM, Université de Toulouse, INRA, CNRS, Castanet-Tolosan, France; University of Toronto, CANADA

## Abstract

Bacterial pathogenicity relies on a proficient metabolism and there is increasing evidence that metabolic adaptation to exploit host resources is a key property of infectious organisms. In many cases, colonization by the pathogen also implies an intensive multiplication and the necessity to produce a large array of virulence factors, which may represent a significant cost for the pathogen. We describe here the existence of a resource allocation trade-off mechanism in the plant pathogen *R*. *solanacearum*. We generated a genome-scale reconstruction of the metabolic network of *R*. *solanacearum*, together with a macromolecule network module accounting for the production and secretion of hundreds of virulence determinants. By using a combination of constraint-based modeling and metabolic flux analyses, we quantified the metabolic cost for production of exopolysaccharides, which are critical for disease symptom production, and other virulence factors. We demonstrated that this trade-off between virulence factor production and bacterial proliferation is controlled by the quorum-sensing-dependent regulatory protein PhcA. A *phcA* mutant is avirulent but has a better growth rate than the wild-type strain. Moreover, a *phcA* mutant has an expanded metabolic versatility, being able to metabolize 17 substrates more than the wild-type. Model predictions indicate that metabolic pathways are optimally oriented towards proliferation in a *phcA* mutant and we show that this enhanced metabolic versatility in *phcA* mutants is to a large extent a consequence of not paying the cost for virulence. This analysis allowed identifying candidate metabolic substrates having a substantial impact on bacterial growth during infection. Interestingly, the substrates supporting well both production of virulence factors and growth are those found in higher amount within the plant host. These findings also provide an explanatory basis to the well-known emergence of avirulent variants in *R*. *solanacearum* populations *in planta* or in stressful environments.

## Author Summary

Metabolic versatility is a critical element for pathogen’s virulence and their ability to survive in the host. Beyond the necessity to collect resources during infection, pathogens face a resource allocation dilemma: they have to use nutritional resources to proliferate inside the host, and in the other hand they need to mobilize matter and energy for the production of essential virulence factors. In this study, we provide evidence of that such a trade-off constrains antagonistically bacterial proliferation and virulence in the bacterial plant pathogen *Ralstonia solanacearum*. We determined the energetic cost required by *R*. *solanacearum* to produce and secrete exopolysaccharide, which is a major virulence factor required for wilting symptom appearance. We validated this result by showing that bacterial mutants defective for exopolysaccharide production or other virulence factor indeed have an increased growth rate compared to the wild-type strain. We provide evidence that this trade-off mechanism is orchestrated by the *phcA* master regulatory gene, which directly connects quorum-sensing regulation to metabolic versatility and virulence. Our results also support the view that *R*. *solanacearum* specializes towards a restricted number of substrates used during *in planta* growth.

## Introduction

Studies in a number of bacterial pathogens in recent years have made it increasingly clear that the ability to assimilate nutrients in the course of host infection is crucial for pathogenesis [1–5]. Pathogens are known to specifically employ amino acid and sugar transporters to gain access to nutrients [6–7] and may subvert the host cell metabolism to re-orientate metabolic fluxes for its own purpose [8–10]. Consequently, the term of ‘nutritional virulence’ has emerged to describe the increasing evidence that, in addition to metabolic adaptation, specific virulence mechanisms enable pathogens to exploit host resources [11].

Metabolic potential (*i*.*e* versatility) is considered a critical element governing a pathogen’s virulence as well as its ability to survive in its host [12]. Beyond the necessity to collect resources within their host, pathogens face a resource allocation dilemma. In one hand they have to use nutritional resources to proliferate inside the host, and in the other hand they need to mobilize matter and energy for the production of essential virulence factors. Indeed, production and secretion of virulence factors are key steps of many infectious strategies to overcome the host immune system [13–14], subvert the host metabolism [8, 15–16], and/or kill the host and succeed in transmission [17]. The resource allocation trade-off is well documented in bacteria [18]: it has been shown for example to occur in the survival / multiplication balance under stress conditions [19] and there is evidence that bacterial growth strategies are the result of trade-offs in the economy of the cell [20–21]. However, this aspect has been poorly studied in the case of pathogens which have to simultaneously acquire nutrients, multiply and produce costly virulence factors in a stressful host environment. Although it is logical to presume that many pathogens experience a resource allocation trade-off to maintain both the proliferation (growth) and the virulence factor production traits during infection, the quantification of the cost for virulence is not documented.

The present study was aimed at understanding how bacterial metabolism supports simultaneously the burden of proliferation and the production of a broad array of virulence factors in the plant pathogen *Ralstonia solanacearum*. *R*. *solanacearum* strains belong to the beta class of Proteobacteria and collectively represent one of the most destructive plant pathogens worldwide due to their unusual wide range of host plants, long persistence in soil and water environments and their broad geographical distribution [22]. Cytological studies have shown that the bacterium invades plants through root wounds and rapidly colonizes the xylem vessels, where it multiplies extensively and produces large amounts of exopolysaccharide (EPS) [23–24]. EPS accumulation in the vascular system and the ensuing collapse of the water flow causes the wilting symptoms and eventually plant death. Expression of virulence factors in *R*. *solanacearum* is controlled by a sophisticated, multicomponent regulatory network that responds to environmental conditions, the sensing of host cells, and bacterial density [25]. More than 20 genes encoding transcriptional regulators, transmembrane sensors or signaling components have been described to control many virulence determinants such as EPS production, plant cell wall degrading enzymes, phytohormones or the Type III secretion system, and virulence-associated functions such as twitching or swarming motility. Much has been learned about how this virulence network functions in culture, but we still have few insights on the coordinated processes that occur during pathogenesis [26–27].

In this study, we used a genome-scale level approach to identify how the allocation of nutritional resource is orchestrated at the molecular level in the context of plant infection when virulence factors are abundantly produced. We conducted a genome-scale reconstruction of the metabolic network of *R*. *solanacearum*, together with a macromolecule network module, including many secreted virulence determinants, which could be used for constraint-based modeling [28–29]. By coupling modeling and experimental approaches, we provide evidence of a trade-off between the expression of growth-supporting pathways and virulence factors. This trade-off mechanism is controlled by the regulatory protein PhcA in a quorum-sensing depending fashion. By using metabolic flux analysis, we show that the cost for virulence factor production in *R*. *solanacearum* strongly impacts bacterial growth and can restrict the metabolic versatility of the pathogen in specific environmental conditions.

## Results

### Reconstruction of the genome-scale metabolic network and a macromolecule network involved in *R*. *solanacearum* virulence

**Fig 1:**
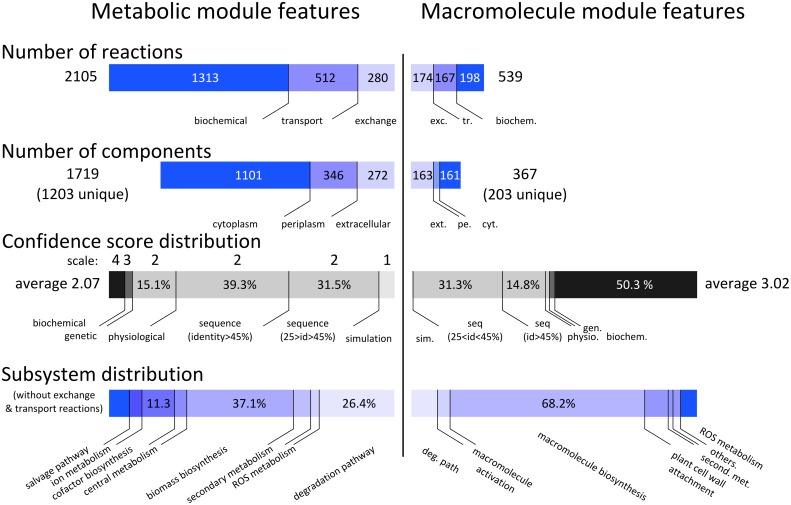
Properties of the *R*. *solanacearum* biochemical reaction network. Compositions and metrics of the genome-scale metabolic network module (left side) and the macromolecule network module (right side). Confidence score corresponds to the evidence of the biochemical reaction included in the network ranging from 0 (low confidence) to 4 (high confidence) as defined by Thiele and Palsson [31].

We generated a metabolic reconstruction consisting of the chemical reactions that transport and interconvert metabolites in *R*. *solanacearum* strain GMI1000. This network reconstruction was achieved through the development of a bioinformatic pipeline (see Material and Methods and S1 Material for details) based on the functional annotation of the genome [30], literature and database searches, and a manual curation protocol [31]. The reconstructed genome-scale metabolic network of strain GMI1000 encompasses 1825 biochemical reactions as well as 280 exchange reactions with the environment linking 1203 unique metabolites localized in three distinct compartments (cytoplasm, periplasm, extracellular). The gene to protein to reaction (GPR) association network, which describes the logical relationship between the genes and the catalyzed chemical reaction, includes 1206 open reading frames. The general features of the metabolic reconstruction are displayed in Fig 1. The full list of genes, metabolites, reactions and GPRs in the metabolic network can be found in S1 Table. The cell biomass composition was both determined experimentally and collected from bibliography (S2 Table).

**Fig 2:**
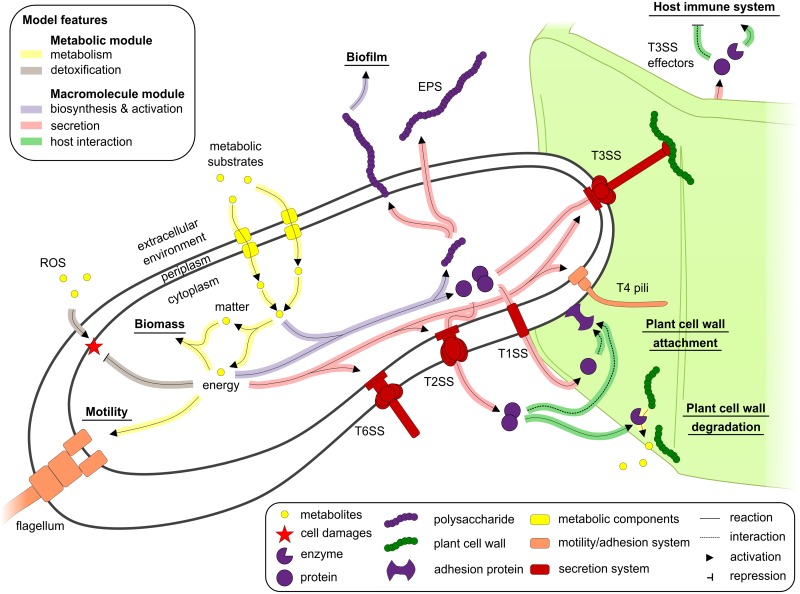
Overview of the bacterial traits included in the genome-scale model of *Ralstonia solanacearum*. Major biological functions included in the macromolecule module of the reconstructed model are displayed. ROS: Reactive Oxygen Species; T1SS, T2SS, T3SS and T6SS: protein secretion system of type I, II, III and VI, respectively.

We added to this reconstructed metabolic network a large subset of reactions involved in biosynthesis, activation and secretion of macromolecules, based on a large body of experimental data. Most of these secreted macromolecules are well-known extracellular virulence factors such as plant cell wall-degrading enzymes, extracellular polysaccharides or pathogenicity effector proteins [25], see Fig 2. This macromolecule module, thereafter called macromolecule network, encompasses 365 biochemical and transport reactions, in addition to 174 exchange reactions with the environment (Fig 1). Among biochemical reactions, 135 correspond to macromolecule biosynthesis reactions and 165 are specifically devoted to secretion processes (Fig 1, S1 Table). Because biosynthesis of macromolecule consumes substrates present within the metabolic network, the macromolecule network and the metabolic network were grouped in a global biochemical reaction network called iRP1476. In total, 109 bibliographic references support the presence of biochemical reactions in the biochemical model (S1 Table). The global model was converted into Systems Biology Markup Language (SBML) format suitable for constraint-based computing. It is available from S2 Material or can be downloaded on the website: http://lipm-bioinfo.toulouse.inra.fr/systemsbiology/models/rsolanacearum.

### Prediction and validation of metabolic phenotypes

**Fig 3:**
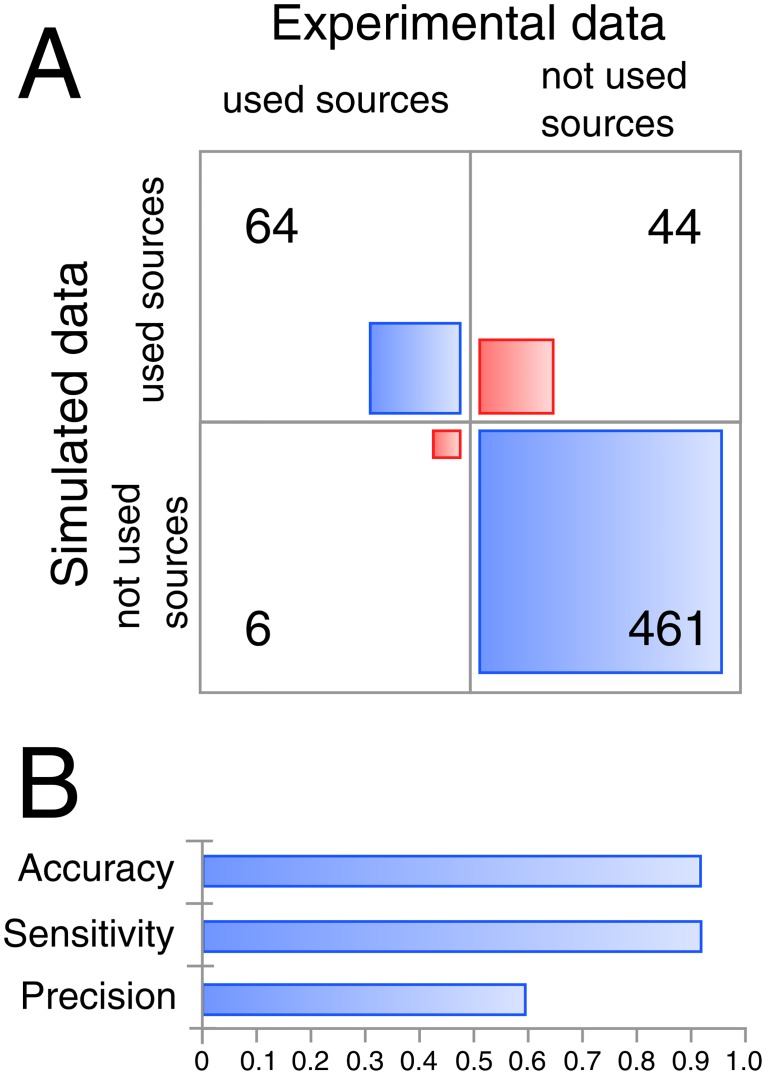
Performance of the model in predicting the substrate usage capacity of *R*. *solanacearum* GMI1000. **(A)** Contingency table of the simulated and experimentally determined metabolic capacities of strain GMI1000 using phenotype microarray (Biolog, PM1, 2, 3, 6, 7, 8). **(B)** The model performance was assessed by calculating the accuracy, the sensitivity and the precision. Sensitivity is the number of true positive on the number of experimentally positive, Precision is the number of true positive on the simulated positive and accuracy corresponds to the overall well predicted phenotypes.

In order to evaluate the performance of the biochemical reaction module in predicting the metabolic versatility of *R*. *solanacearum*, we first determined experimentally the global metabolic capacities of strain GMI1000 using Biolog phenotype microarrays (see Material and Methods). We tested 864 environmental conditions with various Carbon, Nitrogen, Phosphorus and Sulfur sources. This included 190 carbon substrates, and 24 of them were also tested for promoting growth when supplemented to a minimal medium (S1 Fig, S3 Table). The results revealed the usage of 36 carbon substrates by *R*. *solanacearum* (Fig 3A, S3 Table). Dipeptides and nucleotides were neither significantly used as carbon substrates nor as nitrogen sources. Results from phenotype microarray assay were then compared to the model predictions using Flux Balance Analysis (FBA) [32] which calculates the feasibility of cell growth under the different environmental constraints (see Material and Methods). The accuracy of the model prediction was 91.3% over 576 phenotypes, covering 91.4% of the substrates used by strain GMI1000 (Fig 3B). However, 40% of all substrates predicted to be used by the model were not validated in the tested experimental conditions. A similar discrepancy was observed when comparing the results anticipated from the biochemical reaction network with the *in vitro* growth experiments (81.8% of precision). Hence, some metabolites appeared to be not used by strain GMI1000 although the corresponding transporters and the catabolic pathways were predicted to be present from the genome annotation. This observation was suggestive of a potential catabolite repression operating under the conditions tested.

### Evidence for a trade-off between bacterial growth and EPS production

**Fig 4:**
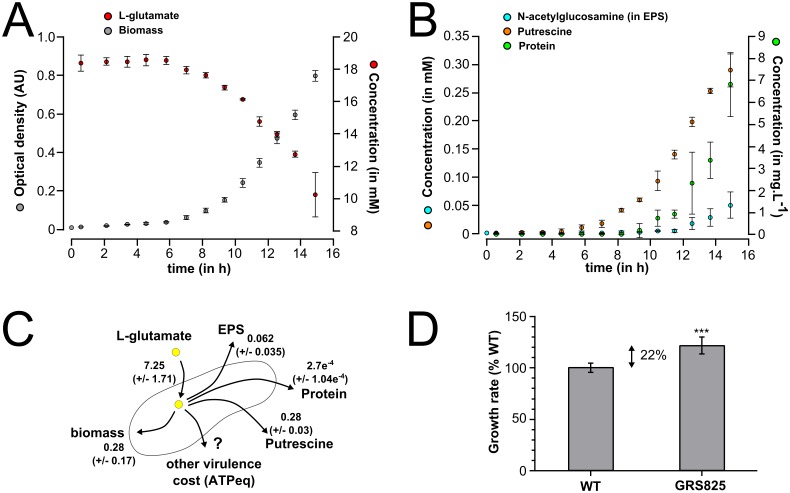
Flux balance analysis of *R*. *solanacearum* growing in minimal medium: evidence for an EPS/biomass production trade-off. **(A**) Kinetics of L-glutamate consumption measured by NMR in batch culture of *R*. *solanacearum* GMI1000 growing in minimal medium. **(B)** Kinetics of EPS, putrescine and total protein content released in culture supernatant. Error bars of panels A and B are 2*σ from 3 biological replicates. **(C)** Flux values in mmol·g_(CDW)_^-1^·h^-1^ (metabolites uptake and production rate and protein) and h^-1^ for the growth rate determined experimentally. The 2 time standard deviation of the value is given under bracket from 3 biological replicates. **(D)** Growth rate of the EPS-defective mutant strain GRS825 compared to wild-type strain in microplates containing minimal medium supplemented with L-glutamate. Significance level (Student-test): ***, p-value <0.001, n = 9.

To validate the reconstructed model quantitatively, we compared the maximal growth rate predicted using FBA with experimental data. For this purpose, we monitored the rate of L-glutamate consumption (Fig 4A) and release of compounds in the medium by ^1^H nuclear magnetic resonance or release of macromolecule using biochemical assays. The kinetics of molecule secretion in the supernatant of cell cultures in minimal medium are shown in Fig 4B and detailed in S4 Table: we found the polyamine putrescine to be strongly produced (S2 Fig), as well as EPS and proteins. The predicted maximal growth rate of the wild-type strain (0.439 h^-1^) was found to be 57% higher than the experimentally measured value (0.280 h^-1^ ± 0.017, 2*σ) based on the amount of consumed L-glutamate (S4 Table). We therefore reasoned that this observed lower growth rate could be due to the metabolic cost for the secretion of virulence factors which adds up to the biosynthesis cost already included in the FBA analysis (Fig 4C). We first investigated the cost for EPS production because EPS is known to be abundantly produced by *R*. *solanacearum in planta* when bacteria reach high cell density [27, 33]. Simulations performed through FBA revealed that the expected gain of growth rate of an EPS-defective mutant was 0.012 h^-1^, which is only 4.2% higher than the wild-type strain (0.280 h^-1^). However, a more important difference was observed experimentally: we created a Δ*eps* mutant impaired for EPS production and found that at high cell density (*i*.*e* above 10^7^ cells/ml) in minimal medium, this *eps* mutant had a growth rate significantly higher (22% ± 6 CI_(95%)_) than the wild-type (Fig 4D). The difference between the predicted cost for EPS biosynthesis and the global cost experimentally measured could be attributed to the energetic cost of EPS secretion. This cost was estimated to be around 8,5 mmol_(ATPeq)_·g^-1^·h^-1^ in ATP equivalent. It cannot be excluded that this estimation includes additional indirect cost for EPS production in addition to its secretion. These results indicated that EPS biosynthesis and its secretion in the environment already represent a significant cost for the pathogen which has a clear impact on bacterial growth.

### A *phcA* regulatory mutant has an optimal growth rate, higher than the wild-type strain at high-cell density

**Fig 5:**
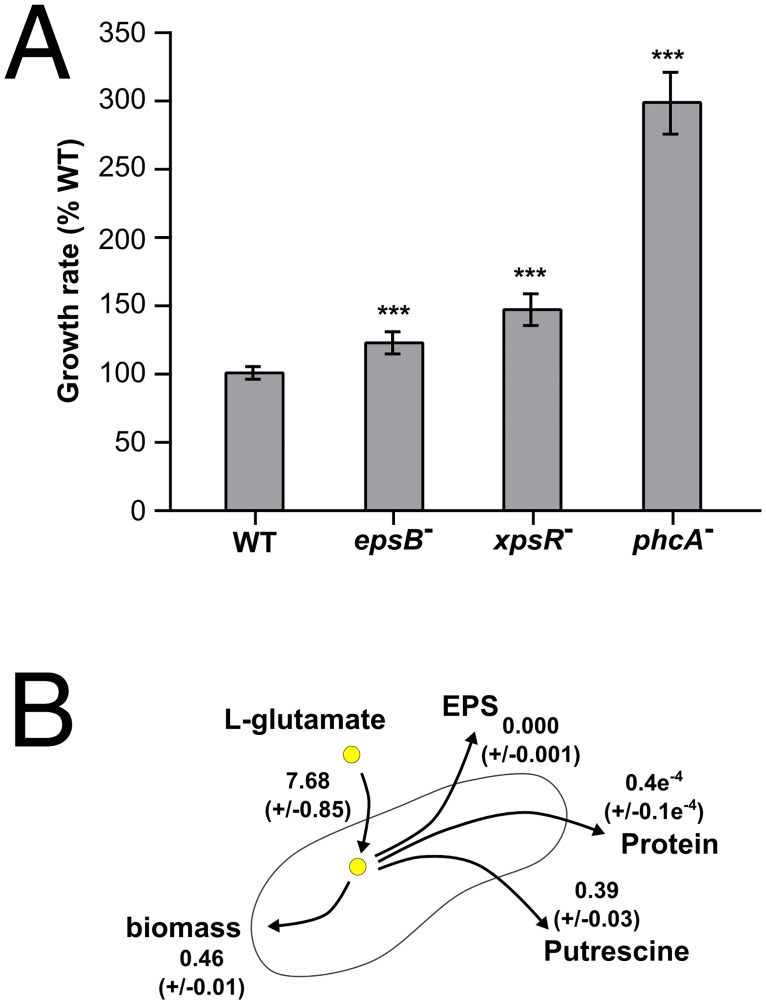
*xpsR* and *phcA* virulence regulatory mutants display a higher growth rate than the wild-type strain. **(A)** Growth rate of the strains GRS825 (Δeps), GRS574 (*xpsR* mutant) and GMI1605 (*phcA* mutant) compared to wild-type in microplates containing minimal medium supplemented with L-glutamate. Significance level (Student-test): ***, p-value <0.001, n = 9. **(B)** Flux values experimentally determined for the *phcA* mutant.

The ‘cost for virulence’ hypothesis was further assessed by monitoring the growth rate of the *phcA* and *xpsR* regulatory mutants. *xpsR* encodes a regulator acting as a downstream cascade component required for activation of EPS biosynthesis [34]. *phcA* encodes a global phenotypic switch regulator under the control of a specific quorum sensing system [35]. PhcA is known to indirectly regulate the production of many virulence factors including EPS via *xpsR*, plant cell wall degrading enzymes and the type III secretion system [25]. At high cell density, the maximal growth rate of the *xpsR* mutant was significantly higher than the one of the wild-type strain (47% ± 8 CI_(95%)_) and the *eps* mutant (20% ± 6 CI_(95%)_), see Fig 5A. An even sharper increase was observed with the *phcA* mutant since its measured maximal growth rate was 198% ± 15 CI_(95%)_ higher than the wild-type. Accordingly, competition experiments conducted in complete medium using an initial 1:1 ratio of the wild-type strain and the *phcA* mutant revealed that after only four hours the fitness of the *phcA* mutant was significantly higher than the wild-type (S3 Fig).

We then performed FBA with the *phcA* mutant grown in minimal medium to determine if the spectacular increased growth rate of this strain was due to an increased substrate consumption rate or to a rerouting of the metabolic fluxes from virulence factor production toward growth. We monitored a similar consumption rate of L-glutamate for the *phcA* mutant (7.68 mmol·g^-1^·h^-1^ ± 0.85 2σ) compared to the wild-type strain (7.25 mM·g^-1^·h^-1^ ± 1.71 2σ) (p-value 0.57). Based on the rate of L-glutamate usage and measured exchange fluxes, the optimal growth rate of the *phcA* mutant was calculated to be 0.435 h^-1^, and found to be close (only 6% deviation) from the measured growth rate, 0.46 h^-1^ ± 0.01 (2*σ) (Fig 5B). This good match between the predicted and observed growth rate further supported the view that the *phcA* mutant had optimal metabolic capacities to sustain growth due to the absence of a cost for virulence factor production/secretion, contrary to the wild-type.

Finally, we inferred from the metabolic flux analysis of the wild-type strain the overall cost of virulence factor production dependent on PhcA. To do so, we optimized an ATP hydrolyzing flux using FBA by setting the biomass production and the L-glutamate consumption rates determined for the wild-type. We found that the cost (in ATP equivalent) for the production of virulence factors controlled by PhcA corresponds to the significant amount of 38.9 mmol_(ATP)_·g^-1^·h^-1^. This amount of energy is indeed comparable to the amount of ATP (34.10 mmol·g^-1^·h^-1^) generated to supply biomass biosynthesis in the *phcA* mutant.

### The rerouting of metabolic resources from proliferation toward virulence factor production is quorum-sensing dependent

**Fig 6:**
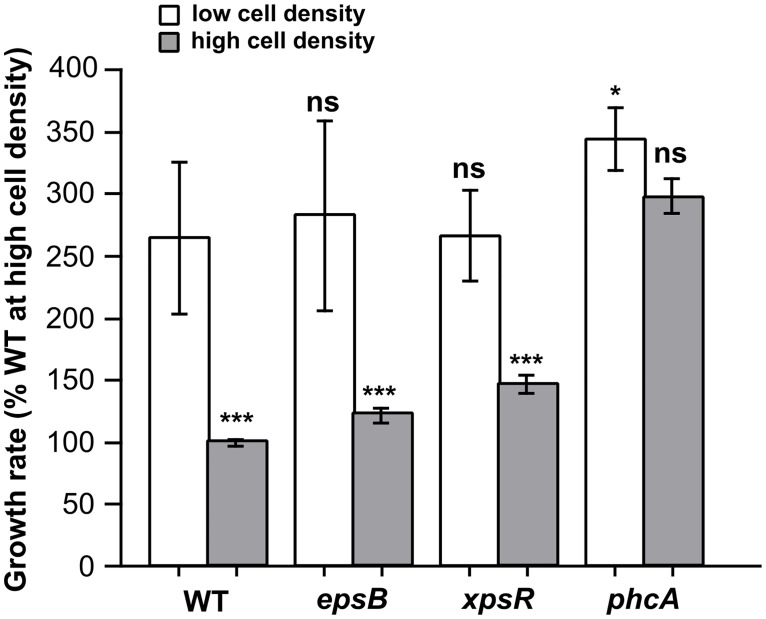
The restriction of growth rate observed for the wild-type strain compared to regulatory mutants is cell-density dependent. Growth rate of the strains GRS825 (Δ*eps*), GRS574 (*xpsR* mutant) and GMI1605 (*phcA* mutant) compared to the wild-type strain at low cell density (white bars) and high cell density (grey bars). Cells were cultivated in microplates containing minimal medium supplemented with L-glutamate. Errors bars are 2*σ (95% data dispersion), n = 6 to 9 biological replicates. Statistical differences were tested in comparison with the wild-type value at low cell density; significance level (Student-test): ns, not significant; *, p-value <0.05; **, p-value <0.01; ***, p-value <0.001.

The metabolic activity of the *phcA* mutant appeared to be focused toward proliferation with a specific usage of resources to sustain optimal growth. Because the activation of PhcA is under the control of a quorum-sensing system, we hypothesized that the wild-type strain should have a similar optimal growth rate at low cell-density (*i*.*e* when PhcA is inactive, as in the *phcA* mutant). Therefore, we monitored the growth kinetics of the wild-type and the *eps*, *xpsR* and *phcA* mutant strains from low cell-density to high-cell density (Fig 6). The measured growth rate for the *phcA* strain at low cell density was in the same range than at high cell density. However, the growth rate of the wild-type strain at low cell density was similar to that of the *phcA* mutant at high cell density (p value = 0.28), thus confirming that the cost for the production of virulence factors which reduces the growth rate of the wild-type strain at high cell density is relieved at low cell density.

### *R*. *solanacearum* metabolic versatility is restricted at high cell density and is under the control of PhcA

The FBA performed above used L-glutamate as sole carbon source. L-glutamate is abundant in the xylem and apoplasm of the tomato host [36] and supports a strong growth of the bacteria in minimal medium. However, if a substrate does not support a strong proliferation rate due to a low efficiency of the corresponding catabolic pathways or a low substrate uptake rate, the cost for virulence factor production might impair bacterial proliferation. We defined a ‘substrate usage capacity’ value that corresponds to the quantification of a phenotypic trait (such as proliferation or virulence) that the bacterial cell produces from a given substrate upon a period of time (for details see S3 Material). Model simulation then showed that below a certain threshold of substrate usage capacity, the expression of virulence functions prevents bacterial proliferation (S4 Fig). This suggested that the trade-off relationship between these two traits is strongly dependent on the nature of the resources collected in the environment.

**Fig 7:**
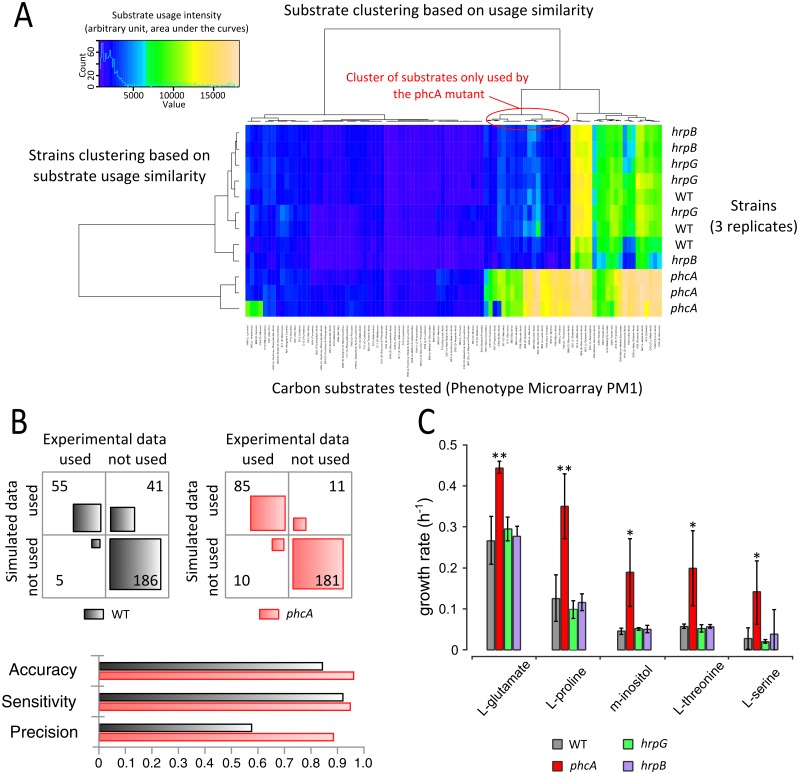
Virulence factor production restricts the versatility in a *phcA*-dependent manner. **(A)** Heatmap of the phenotype microarray data (plate number 1, PM1) of strain GMI1000 (wild-type) and the regulatory mutants GMI1605 (*phcA*), GMI1755 (*hrpG*) and GMI1525 (*hrpB*). The clustering is performed depending on the similarity of substrates usages. **(B)** Maximum growth rate of the wild-type strain and the mutants in minimal medium supplemented with different carbon sources. Errors bars are 2*σ (95% data dispersion), n = 6, significance level (Student-test): *: p-value <0.05; **: p-value <0.01. **(C)** Contingency table of the simulated and experimental metabolic capacities of the wild type strain and the *phcA* mutant determined using phenotype microarray (PM1, 2, 3). The model performance is reported as precision, sensitivity and accuracy for the two data sets.

In order to explore whether the proliferation is impacted by virulence factors production upon usage of various nutritional resources, we determined the metabolic profile of the *phcA* mutant and two other strains defective for major virulence transcription factors (*hrpB* and *hrpG*). *hrpB* encodes the downstream regulator of the Type III secretion system and dependent substrates, and *hrpG* encodes a plant signal-responsive coordinator of multiple pathogenicity functions [37]. Microarray phenotyping revealed that the metabolic profile of the *hrpB* and *hrpG* mutants was similar to those of the wild-type strain whereas the *phcA* mutant displayed remarkable expanded versatility (Fig 7A and S5 Fig, S5 Table). Indeed, the *phcA* mutant possessed a wider substrate usage than the wild-type strain, being able to catabolize 17 additional substrates to sustain proliferation. For example, L-proline, myo-inositol and L-serine were significantly used as carbon substrates only by the *phcA* mutant strain (p-value 4.0e^-6^, 2.7e^-5^, and 5.0e^-5^, respectively). Comparison of the versatility predicted by the biochemical reaction network and the versatility experimentally observed using the *phcA* mutant indicated a high accuracy of the model prediction (Fig 7B). Indeed, the precision increased from 57% with the wild-type strain to 89% with the *phcA* mutant.

To confirm the effect of the *phcA* mutation on metabolic versatility observed using substrate microarrays, we monitored bacterial growth of the *hrpB*, *hrpG* and *phcA* mutants in minimal medium supplemented with five substrates found to be differentially metabolized (p-value<0.01). Results shown on Fig 7C indicate that the *phcA* mutant had a significantly enhanced growth rates (p-value<0.01) on these five carbon substrates, indicating that not only usage of additional substrates but also quantitative increase of the usage of several substrates is dependent upon PhcA. Altogether these observations confirmed that the metabolic network is optimally oriented toward proliferation in the *phcA* mutant.

### Flux balance analysis identifies the critical substrate usage capacity threshold required for *R*. *solanacearum* fitness

**Fig 8:**
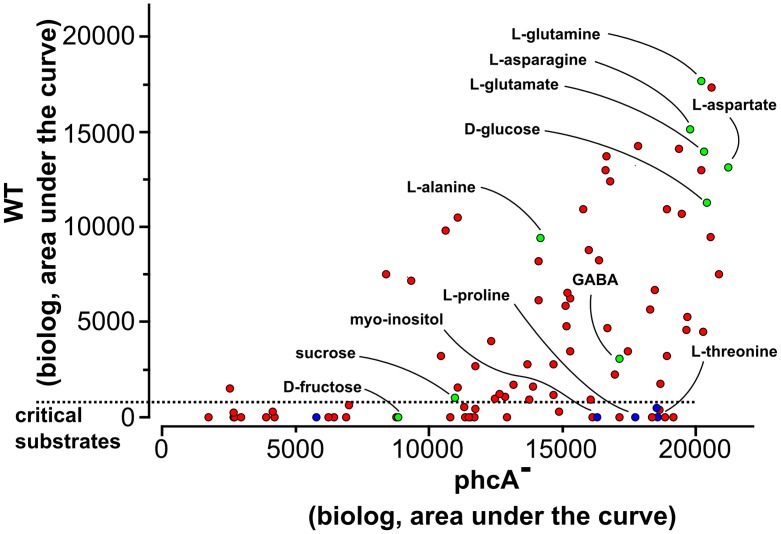
Cost of virulence factor production impairs growth on critical substrates. Comparison of the substrate usage capacity inferred from biolog phenotype microarray between the wild-type strain and the *phcA* mutant and its ability to support bacterial proliferation. Labeled compounds are carbon substrates known to be present *in planta* (xylem or apoplast) at a concentration >90 μM (in green) or lower concentration (in blue) [36]. Critical substrates which do not sustain a bacterial growth <5% of the maximal substrate usage capacity are those located below the dashed line.

In order to determine if the observed reduced versatility was dependent on the metabolic cost of virulence factor production, we performed a FBA through the reconstructed genome-scale model. Previous FBA results indicated that the *phcA* mutant and the wild-type strain had a similar substrate uptake rate in presence of L-glutamate, a substrate which supports growth of both strains. We therefore estimated the minimal consumption rate of various substrates supporting the growth of the *phcA* mutant (such as L-serine, L-proline, L-threonine, sucrose, D-fructose, D-glucose and myo-inositol). The uptake rate for seven tested substrates was estimated in the range of 0.91 (for sucrose) to 6.24 mmol.g^-1^.h^-1^ (for L-Serine). The correlation (R² 0.53, see S6 Fig) between the substrate uptake rates (in C-mole) with the growth rates observed experimentally indicates that the difference in substrate usage capacity of the *phcA* mutant does not only rely on difference in uptake of substrates but also in the efficiency of metabolic pathways used for their assimilation. Then, we performed FBA simulations using as additional constraint the cost for virulence factor production (as determined previously in ATP equivalent). For all tested substrates, the optimal growth rate predicted through FBA matched remarkably well (R² 0.80) with the one monitored experimentally (S7 Fig). This analysis also revealed that certain carbon substrates (those supporting a growth rate of the *phcA* mutant below 0.15 h^-1^) are unable to support bacterial growth of the wild-type strain since their metabolic conversion into biomass cannot be realized due to the imposed cost for virulence factor production. This explains why certain carbon sources such as L-serine and D-fructose, as well as compounds just above the threshold in liquid culture like L-threonine and myo-inositol, do not support the wild-type strain proliferation on the phenotype microarray (Fig 8). Hence, a fixed cost of virulence factor production satisfactorily explains in most cases the observed reduction of versatility in the wild-type strain and allows defining (i) a critical substrate usage capacity threshold around 0.15 h^-1^, and (ii) a list of substrates which are not enough metabolized to promote substantial growth beyond this threshold (see S6 Table).

## Discussion

A first achievement of this study was the reconstruction of a *R*. *solanacearum* genome-scale cell model integrating knowledge collected on this microorganism over the last 40 years. With more than 2644 reactions manually curated, this reconstruction is in the range of the highest standards for bacterial models [32, 38–39]. Moreover, this reconstruction associates a metabolic network with a macromolecule network to account for the production and secretion of virulence factors since *R*. *solanacearum* is known to produce hundreds of extracellular proteins involved in pathogenesis [40–41]. *R*. *solanacearum*, as many other bacteria in the *Burkholderiales* order, can adapt to many different habitats and host plants [42–43]. These bacteria generally possess a large genome (> 5.5 Mb) with a significant proportion of variable genes which are presumably involved in adaptive responses to changes in environment, permitting the bacteria to thrive in diverse ecological niches [44–46]. The presence in the reconstructed model of specific pathways devoted to plant pathogenesis (such as phytohormone biosynthesis, Type III secretion or EPS production) provides a first estimate of the functional overlap between the bacterial metabolism and the establishment of the pathogenicity program. In addition, this first description of *R*. *solanacearum* versatility through a fine mapping of the used metabolic substrates opens the way to a global correlative analysis of the trophic abilities of the bacterium and its colonization capacity of a broad set of environmental niches including a large host spectrum.

Flux balance analyses using the reconstructed model along with the measurements of the metabolites/macromolecules uptake and secretion rates unexpectedly revealed that the wild-type strain, when grown under non-limiting nutrient availability (*i*.*e*. batch culture in minimal medium), had a significantly restricted growth rate when it exceeds the threshold of 10^7^ cells/ml. The measured growth rate at high cell density is indeed around 60% lower than the one predicted by simulations when considering the full metabolic potential inferred from the reconstructed *R*. *solanacearum* model. We found that this optimal growth rate of the wild-type strain can be reached at low cell density and that the growth rate decrease observed above 10^7^ cells/ml is therefore dependent upon a quorum-sensing mechanism. We have shown that this growth rate restriction is dependent on the *phcA* gene, a master regulator controlling multiple virulence traits of the pathogen [47]. As expected from the behavior of the wild-type strain at low cell density, we found that the growth rate of the *phcA* mutant is more than 60% higher than the wild-type strain at high cell density, while it approximates the optimal growth rate predicted through model simulations (6% deviation). *phcA* is known to control a phenotypic switch from non-mucoid to mucoid EPS-producing colonies in response to cell density [35, 48] and *in vitro* expression studies have shown that, in addition to EPS, this gene controls multiple virulence functions including the production and secretion of plant cell wall degrading enzymes, flagellar motility, twitching motility, siderophore production or the Type III secretion system (reviewed in [25]). Accordingly, a *phcA* mutant is unable to cause disease symptoms when inoculated on plants [47]. Constraint-based modeling was used to predict and quantify the cost of the huge EPS production observed in *R*. *solanacearum*. These results were confirmed by monitoring growth rates of *eps* or *xpsR* mutants, and we showed that EPS biosynthesis indeed represents a significant cost for bacteria but this remains a marginal value compared to the growth rate gain observed in the *phcA* mutant. This strong phenotype certainly results from the pleiotropic nature of the *phcA* mutation since this gene orchestrates a so-called ‘phenotypic conversion’ [47] and controls multiple virulence functions encoded by hundreds of genes, including *xpsR* and the *eps* gene cluster. Future determination of the PhcA regulon will provide clues on the additional biological functions which may represent a significant cost for the cell. This approach should also reveal the probable rewiring of the PhcA-downstream regulatory network which occurs in the mutant.

The picture emerging from these results is the existence in *R*. *solanacearum* of a clear trade-off between functions dedicated to proliferation (bacterial growth) and functions required to produce virulence factors. Bacteria use a complex regulatory network to organize the preferential allocation of metabolic resources to growth or virulence functions depending on the cell density status. PhcA appears to be the key regulatory component that governs this developmental switch occurring when bacterial populations reach 10^7^ cells/ml. This trade-off implies that this pathogen, despite its huge multiplication in plant xylem vessels, doesn’t need to grow fast as theoretically possible to achieve a successful infection. It also highlights that bacterial virulence and metabolism are intertwined and illustrates how resource allocation is a critical mechanism with a profound impact on pathogenic fitness. Diverse range of growth/virulence balances have been described among various pathogens [49]. For instance S*almonella* deals with the trade-off between a fast growth in order to outcompete commensals or defective variants and the production of the Type III secretion system required to complete infection [50–51]. Another strategy is to delay the massive production of a virulence factor until the success of host colonization [49, 52]. As observed for *R*. *solanacearum*, these traits are often under a social control system, such as quorum sensing or environmental stimuli, to ensure the coordination of the costly production of the virulence toxin [49].

We discovered that the *phcA* mutation had also a dramatic impact on the *R*. *solanacearum* metabolic versatility. The *phcA* mutant strain is indeed able to metabolize 17 carbon substrates that the wild-type strain is unable to use to support its growth. In addition, the *phcA* mutant has an increased ability to use many other substrates, better than the wild-type. Interestingly, this increased substrate usage pattern matches well (63%) with the list of substrates identified in the apoplasm and xylem fluids of the host plant tomato [36]. For example, many amino acids, including L-proline and L-serine, which are present in xylem and apoplastic tissues, enable growth of the *phcA* mutant but not the wild-type strain.

**Fig 9:**
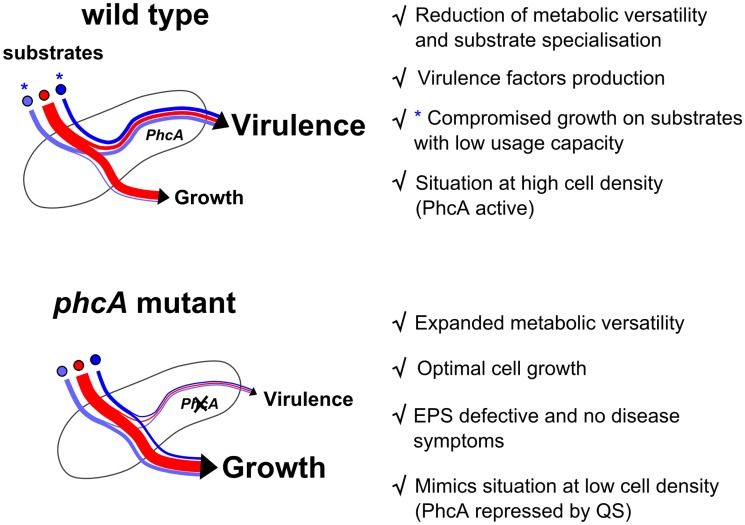
A summary of the PhcA-dependent growth/virulence trade-off in *R*. *solanacearum*.

Rather paradoxically, these results indicate that metabolic versatility is reduced when PhcA is active, *i*.*e* at high cell density and so presumably at the onset of the massive plant colonization. The growth/virulence trade-off hypothesis can explain this reduction of versatility as evidenced by FBA. However, it cannot be excluded that PhcA also negatively controls the expression of several metabolic transporters or catabolic pathways. On many substrates, *R*. *solanacearum* harbors a low substrate usage capacity and those substrates are unable to sustain efficient bacterial growth when PhcA is active. But the pathogen has also a clear nutritional preference towards certain compounds that are abundant *in planta* such as L-glutamine, L-glutamate and D-glucose, and which promote strong bacterial growth. The compounds detected in low amount in tomato fluids (<90 μM) were associated with a low substrate usage capacity by the wild-type strain (chi-test p-value 4·10^−7^) whereas the used compounds correlated with the range of the most abundant compounds *in planta* (p-value 0.033). Hence, it is tempting to speculate that *R*. *solanacearum* specialized to preferentially metabolize those prominent substrates in plant tissues, especially when it reaches high cell density in xylem vessels. This hypothesis is supported by the finding that L-glutamate has the higher uptake rate in C-mol by *R*. *solanacearum* over all the compounds tested. This also implies that despite a broad host range and a wide metabolic versatility, this pathogen tends to specialize to relatively few compounds present *in planta*. On the other hand, it suggests that at low cell density (in the soil or at the very early stages of infection) the *phcA*-dependent repression of virulence functions leads to an increased metabolic versatility that could be beneficial in a low-resource and competitive environment [53], see Fig 9.

Altogether, these results also highlight how the maintenance of a pathogenicity trait can be challenging for the pathogen since the existence of this growth/virulence trade-off can lead to the emergence of non-virulent variants with a better growth rate than their wild-type ancestor. Interestingly, it is known for more than fifty years that when inoculated into tomato plants, some members of the *R*. *solanacearum* population spontaneously undergo a phenomenon called ‘phenotypic conversion’ [54]. Phenotypic conversion (PC) was shown to be the consequence of DNA replication errors and transposition of insertion sequence elements that inactivate *phcA* [48, 55]. Recently, serial passage experiments of *R*. *solanacearum* on various hosts over 300 hundred generations also resulted in the occurrence and propagation of PC-type mutants that outcompeted the ancestor strain in several lineages [56], indicating that such mutants can be strongly selected *in planta*. Our results provide an explanatory hypothesis to this well-described occurrence of PC-type variants since such variants (i) do not pay the cost for virulence factor production and thus reach an optimal growth rate, and (ii) escape the restriction of metabolic versatility that takes place at high cell density in the wild-type strain. Both properties provide a clear competitive advantage to the variant in presence of the wild-type strain. Because PC-type mutants do not produce many virulence factors and are unable to cause disease symptoms, this raises the question of whether such variants that do not contribute to the public goods but exploit the resources can be considered as ‘cheaters’ in the infecting population. The fast-growing and highly motile phenotype of *phcA* mutants rather incites to view such variants as ‘colonizers’ when populations face stressful conditions or environments. Interestingly, the reversion of natural PC-type (*phcA*) mutants to the wild-type form after *in planta* multiplication was reported [55], suggesting that the balance in infecting populations between low-growing, virulent bacteria and ‘colonizer’, low-pathogenic variants could have a wider impact on the pathogenic strategy and global life cycle of *R*. *solanacearum*. Future work aimed to study the transmission of the pathogen and its persistence in the environment should provide further clues to evaluate the role of PC-type mutants in dissemination and ecological success of the pathogen.

## Materials and Methods

### *In vivo* experimental procedure and analytics

#### Bacterial strains and growth conditions

*R*. *solanacearum* strains used are the wild-type strain GMI1000 [30] and the following regulatory deletion or disruption mutant derivatives: GMI1525 (*hrpB*::Ω) [57], GMI1755 (Δ*hrpG*) [37] and GMI1605 (*phcA*::Ω) [58]. The EPS-defective mutant was obtained by inserting the Ω interposon strain [59] into the distal *Eco*RV sites located within the *epsB* and *epsD* genes in the pSG837 plasmid from the GMI1000 library [30]; this construct was used to transform *R*. *solanacearum* and a double recombination event was selected using spectinomycin resistance from the Ω cassette. The resulting strain GRS825 carries a 4.35 kb deletion encompassing the *epsB*, *epsC* and *epsD* gene, which was verified by PCR. Strains were grown in complete medium (BG medium) or in minimal medium at pH 6.5 [60] at 28°C. The minimal medium was supplemented with various carbon sources at 20 mM. Liquid cultures for FBA were performed in shake flask containing 150 ml of culture agitated at 180 rpm. Liquid cultures for growth experiments were performed in 96 well-plates containing 200 μl of culture agitated linearly at 600 rpm and monitored using the FLUOstar Omega microplates reader (BMG Labtech). Optical density of the liquid culture was measured at 600 nm every 5 min. Growth experiments at low cell density were inoculated at a cell density of 5·10^5^ cell·ml^-1^. Growth experiments at high cell density were inoculated at a cell density of 5·10^7^ cell·ml^-1^., a value corresponding to the quorum sensing threshold [35].

#### Phenotype microarray

Phenotypic microarrays were performed using Biolog Phenotype Microarray plates, PM 1,2,3,4,6,7,8,9,10, and following the manufacturer’s protocol modified as following. Before inoculation of Biolog fluid IF-0, the cells were collected from a static culture on plate containing agar complete medium (BG medium) and resuspended in sterile H_2_O supplemented with D-glucose at 20 mM. Thus, they were starved for nitrogen, phosphorus and sulfate during 6h at 28°C and 180 rpm agitation. The step of starvation in H_2_O during 6h was found to reduce the background observed in PM 3 and PM4. D-glucose at 20 mM concentration was used as carbon source for the inoculation of the plates 3, 4, 6, 7, 8, 9, 10. The plates were incubated at 6·10^7^ cell·ml^-1^ and measurements were recorded on Omnilog reader (Biolog) during 96 hours. Because the activity of the respiratory chain of bacteria is observed from the reduction of a tetrazolium-based chemical, stained cells can be monitored only if substantial proliferation is obtained. Data were analyzed and statistic calculated using the R software package OPM [61].

#### NMR analysis of bacterial culture supernatants

Exocellular compounds were collected from cultivation broth in shake flasks. A volume between 3 and 10 ml of culture supernatants was collected depending on bacterial concentration, filtered on 0.22 μm filter (Millex syringe filters) and frozen at -20°C until analysis. Metabolites in culture supernatants were quantified by Nuclear Magnetic Resonance (NMR) spectroscopy. 1D ^1^H spectra were recorded on an Avance II 500-MHz NMR spectrometer using a 5-mm z-gradient BBI probe head (Bruker, Rheinstatten, Germany) at a temperature of 298°K. NMR measurements were acquired by using a 30° pulse, 5.000-Hz sweep width, and 3.27-s acquisition times and recording 16 scans with a relaxation delay between scans of 10 seconds. A pre-saturation of the water signal was performed during the relaxation delay. An amount of 100 μl of D_2_O (> 99% Eurisotop) containing 1.6 mM of TSP-D_4_ (3-(trimethylsilyl)-2,2′,3,3′-tetradeuteropropionic acid) was spiked into 500 μl of filtered culture supernatant in order to correct the field drift and performing the quantification of compounds relatively to the TSP-D_4_ quantity. Identification of putrescine was performed by measuring chemical shifts of standard compounds (>98% Sigma-Aldrich).

#### Exopolysaccharide and protein quantifications

EPS quantification in culture broth supernatant was determined by measuring hexosamine content with the Elson Morgan Assay, using a similar protocol to Brumbley et al. [48]. Briefly, EPS in culture supernatant were precipitated in 2.5 ml Eppendorf tubes by adding NaCl at 0.1 mM final concentration, then 4 volumes of acetone were added, and then the samples were stored at 4°C over night. Precipitated EPS pellet were recovered in 200 μl H_2_0 and heated at 65°C on heatblock during 10 min. Insoluble material were discarded after centrifugation at 13 000 rpm during 5 min at 4°C. A volume of 0.15 ml 12M HCl and 0.250 ml H_2_O were added, and hydrolysis of the sugar polymers was performed at 110°C during 30 min with a heatblock. Hydrolyzed samples were cooled down at room temperature. Then, 0.4 ml of 2 M Na_2_CO_3_ was added, homogenized, and subsequently 0.5 ml of 2% Acetyl Acetone in 1.5 M Na_2_CO_3_ was added. The samples were then heated at 100°C with a heatblock for 20 min. After being cooled down at room temperature, the liquid was transferred in 15 ml Falcon tubes and 1.0 ml 95% ethanol (≥99.8% Sigma-Aldrich) was added. Then, 0.5 ml Erlich's reagent solution (0.4 g of para-dimethyl-aminobenzaldehyde (≥98%, Sigma-Aldrich) in 6.0 ml ethanol) was added. Samples have been kept for 30 min in the dark (with aluminum) and then the absorbance at 530 nm has been recorded. Quantification of hexosamine was determined by running standard curve of N-acetyl-galactosamine (>98%, Sigma-Aldrich) which has followed the overall process except precipitation.

Protein quantification in culture supernatant was performed using Bradford essay. 1 ml of Bradford 1X Dye reagent (Sigma-Aldrich) was added to 100 μl sample and absorption was measured at 595 nm after 5 minutes of incubation. Bovine serum albumin was used as standard for the quantification.

#### Competition experiment

The wild type strain and the *phcA* mutant were inoculated in equal proportion (5·10^7^ cells·ml^-1^) in minimal medium supplemented with L-glutamate at sole carbon source. The proportion of each strain was monitored over time by plating serial dilutions of the culture medium on BG plates supplemented with triphenyl tetrazolium chloride which allows easy discrimination of *phcA* mutants (dark red non-mucoid colonies).

### *In silico* experimental procedure

#### Genome-scale metabolic model reconstruction

The reconstruction of the metabolic model of *R*. *solanacearum* was performed following the protocol to generate a high-quality genome-scale network published by Thiele and Palsson [31]. However, the production of the initial draft reconstruction from the genome annotation has been performed following a different process as described below and then the expert manual curation has been performed.

The draft metabolic reconstruction has been set up following the protocol explained below and detail in the S1 Material. Briefly, this draft was obtained by collecting reactions from already reconstructed networks of various organisms based on gene homology with *R*. *solanacearum* strain GMI1000 following three steps. Each step is supported by an automatic or a semi automatic bioinformatics tools. i) The first step of the draft reconstruction was to generate four draft metabolic networks built from four metabolic models from other bacteria, i.e. *Ralstonia eutropha* (RehMBEL1391 [62]), *Bacillus subtilis* (Bs_iYO844 [38]), *P*. *aeruginosa* (iMO1086 [39]), and *Escherichia coli* (iJO1366 [32]). The method used is inspired by the Autograph method [63] (see Section I. B in S1 Material). Briefly, reactions of each bacteria models harboring an associated gene for an orthologous gene in strain GMI1000 filling the following requirement, >30% Identity and >50% coverage, were collected. Gene orthology was assessed using Inparanoid [64] on bacterial proteomes. ii) Then, since these metabolic models use different ontologies, the second step has been to standardize the identifiers of the four draft metabolic models. For accelerating this step, we have developed SAMIR (**S**emi **A**utomatic **M**etabolic **I**dentifier **R**econciliation), a web tool that allows reconciling the identifiers of reactions and metabolites between the different draft models in a semi automatic way (see Section I. C in S1 Material). iii) At last, the four standardized draft metabolic models were merged in a single one following an expert evaluation of the best annotation reactions based on the homology analysis and the evidence of the reactions. This step was facilitated by using a relational database in Access linking common identifiers through the four draft networks (see Section I. D in S1 Material). All the methods and tools used in these three steps are detailed in S1 Material.

Reaction candidates of the high-quality draft reconstruction and genes annotated to be involved in metabolic processes were curated one by one following an expert evaluation of their information. Thus, curation of the reactions was done by seeking for information in metabolic databases KEGG [65] and MetaCyc [66] as well as the transporter database TCDB [67]. On purpose, a *R*. *solanacearum* GMI1000 specific pathway/genome database was generated using the Pathway-tools software [68] to assess their metabolic pathway context, and concomitantly, the MicroScope platform [69] was used to evaluate the genomic context (e.g. shared synteny). In addition, it was used to access easily the various information provided on the platform on genes, like blast through various databases and links to the bibliography for the blast hits, prediction of gene product localization, prediction of EC number. Dead-end reactions were computed thanks to MetExplore [70] and the gap filling was performed by reassessment of pathway context through the previously listed tools and databases. Transport reactions as well as assimilation pathways of various substrates were curated thanks to the analysis of the phenotype microarrays performed with the Biolog technology (see below for detailed protocol). The mass and charge balance of the network were computed and corrected. Stoichiometrically balanced cycles were computed by running flux balance analysis without open exchange fluxes and those leading to free conversion between NADPH and NADH were curated; those which had no consequences on flux simulation results were kept. Finally, the model was converted into Systems Biology Markup Language (SBML) format (S2 Material) suitable for constraint-based computing and was documented following MIRIAM Registry recommendation, with assignment of a Systems Biology Ontology references at each component of the model [71], assignment of metadata like bibliography information and confidence level information. A dedicated webpage which allows downloading the latest version of the model and all additional information are available at this location (http://lipm-bioinfo.toulouse.inra.fr/systemsbiology/models/rsolanacearum. At last, the metabolic network has been added to the MetExplore web site [70], what allows visual exploration and -omics data mapping on the whole network (directly available at http://metexplore.toulouse.inra.fr/metexplore2/index.html?idBioSource=3631)

Biomass composition of the *R*. *solanacearum* cell was assessed from published composition of several biochemical components determined experimentally, or assumed to be similar to *R*. *eutropha* [62] or *E*. *coli*. The cell dry weight (CDW) quantification of strain GMI1000 (0.414 g_(CDW)_·OD^-1^·l^-1^ ± 0.042, 2·σ) and the *phcA* mutant (0.453 g_(CDW)_·OD^-1^·l^-1^ ± 0.072, 2·σ) and the total protein content were determined experimentally as described previously [72] using cultures grown exponentially until an optical density of 0.5 at 600nm in minimal medium supplemented with L-glutamate as sole carbon source. List of biomass composition is available in S2 Table.

#### Macromolecule network reconstruction

The reconstruction of the macromolecule network (macromolecule biosynthesis and secretion, host interaction, and DNA modification) was performed following the same rigorous process established for the metabolic network whether concerning expert curation of each reaction or validation of their stoichiometry, identification of dead-ends as well as gap-filling. The macromolecules included in the model are mainly secreted macromolecules identified from experimental data (bibliography) or genomic information coming from prediction of protein secretion signals using the software PSORTb [73]. Reactions included in the model are the biosynthesis reactions of the macromolecules, transport reactions outside/inside of the bacterial cell, exchange fluxes with the environment and for host-interaction reactions. Cost of secretion, i.e. energy in ATP hydrolysis or proton gradient, was assigned by assuming one ATP hydrolysis for each metabolite unit in the macromolecule transported through one membrane when no information was available. For instance one ATP hydrolysis was considered for each amino acid in a protein, which is transported via the secretion system. Macromolecule interactions in the extracellular space were modeled by condensation reactions where a macromolecule complex is generated from macromolecule substrates. The macromolecule network modules were lumped together with the GS-metabolic network module in a SBML file, see Fig 2. Thus, calculation of the cost of production and secretion of various macromolecules can be assessed by performing flux balance analysis through the whole model.

#### Simulation of the biochemical network

Simulation of the biochemical reaction network state containing the genome-scale metabolic network module plus the macromolecule network module was performed using constraint based modeling [29]. Flux distributions were simulated by Flux Balance Analysis using the software FlexFlux [74]. Various constraints were applied on simulation. Environmental conditions were defined by constraining lower and/or upper bound of exchange fluxes depending of the availability of the substrate. Substrate uptake rates were constrained from experimentally measured fluxes when available or the upper bound was fixed to 5 mmol·g_(CDW)_^-1^·h^-1^ as default value. System outputs like EPS production or putrescine production fluxes were constrained using the experimentally measured fluxes (see S4 Table).

#### Phenotype microarray prediction

Qualitative evaluations of the cell scale model performance in predicting the phenotypes microarray were performed by comparing the model predictions with the experimental measurements. Growth predictions in various environments were performed by *in silico* simulation as described in previous section with optimizing the biochemical reaction corresponding to the biomass function. If the biomass reaction flux is not null the growth is considered as performed by the bacteria and thus the prediction is classified as active (1); if the biomass reaction flux is null the prediction is classified as not active (0). Then, a binary matrix (i*j) containing the contingency of the experimental growth (i) and network prediction growth (j) with the two classes (0, 1) are built and the network performance metrics are calculated as following. The true positive (TP) phenotypes correspond to identical value of 1 for i and j. The false positive (FP) phenotypes correspond to a value of 0 for i and 1 for j. The true negative (TN) phenotypes correspond to identical value of 0 for i and j, and the false negative (FN) phenotypes correspond to a value of 1 for i and 0 for j. The sensitivity (Sn) of the model prediction capacity which corresponds to the true positive rate is calculated as following, where (P) is the number of experimentally active results (i = 1):

$$Sn=\frac{TP}{P}$$

The precision (Pr) which is the positive predictive value is calculated as following:

$$Pr=\frac{TP}{\left(TP+FP\right)}$$

The faux positive rate (FPR) is calculated as following:

$$FPR=\frac{FP}{N}$$

The accuracy (Acc) of the model prediction capacity is calculated as following:

$$Acc=\frac{\left(TP+TN\right)}{\left(P+N\right)}$$

## Supporting Information

S1 Fig Phenotype Microarray curves of *R*. *solanacearum* strain GMI1000. Phenotype Microarray data of strain GMI1000 on Biolog plates PM1 to PM10 incubated during 96h at a temperature of 28°C. The number of replicates is from 3 to 7. Data were treated with the R package opm.
(PDF)

S2 Fig Superimposed ^1^H NRM spectrum of culture supernatant of *R*. *solanacearum* growing on minimal medium with L-glutamate as sole carbon source (blue) and standard putrescine in D_2_O (red). (PDF)

S3 Fig Competition essay between the wild-type strain GMI1000 and the *phcA* mutant strain in minimal medium with L-glutamate as sole carbon source. Liquid cultures were inoculated with an equal proportion of the GMI1000 and the *phcA* mutant, 5·10^7^ cell.ml^-1^. Significance level: ns, not significant; *, < 0.05; **, <0.01.
(PDF)

S4 Fig Theoretical trade-off between proliferation and virulence factors production for varying substrate usage capacity (SUC) of the cell. For each SUC considered the pareto surface, which corresponds to the trade-off surface optimizing both objectives, is drawn. Medium and low SUC were obtained by decreasing substrate uptake rate as yield.
(PDF)

S5 Fig Phenotype Microarray curves of *R*. *solanacearum* strains GMI1525 (*hrpB*::Ω), GMI1605 (*phcA*::Ω), and GMI1755 (Δ*hrpG*). Phenotype Microarray data were collected upon 96h at a temperature of 28°C for the plates PM1, PM2 and PM3. Three replicates were performed. Data were treated with the R package opm.
(PDF)

S6 Fig Minimal substrate uptake rates calculated using FBA supporting the growth rates experimentally determined with different sources of carbon for the *phcA* mutant. The linear correlation (red line) is 0.53.
(PDF)

S7 Fig Comparison of the growth rate of *R*. *solanacearum* strain GMI1000 calculated using FBA with the experimental growth rate observed using different carbon substrates. The linear correlation (black line) is 0.80. The simulated growth rates were calculated by FBA using the minimal substrate uptake rates of the *phcA* mutant plus the cost of virulence factors determined previously as constraints.
(PDF)

S1 Material Supplementary material. Supplementary material containing details on the reconstruction pipelines, and the various *in silico* analyses. Detail of the algorithms used for *in silico* analyses and the corresponding scripts are available and can be freely downloaded at the following location: http://lipm-bioinfo.toulouse.inra.fr/systemsbiology/models/rsolanacearum.
(PDF)

S2 Material Genome-scale Biochemical model iRP1476. Genome-scale biochemical model of *R*. *solanacearum* GMI1000, iRP1476, in sbml format.
(SBML)

S3 Material Definition of the substrate usage capacity. (PDF)

S1 Table Biochemical reaction network. Information on the biochemical reaction network which contains the genome-scale metabolic network and the macromolecule network.
(XLSX)

S2 Table Biomass composition. Biomass composition and energetic calculation of the biochemical reaction network.
(XLSX)

S3 Table Phenotype microarray of *R*. *solanacearum* strain GMI1000 and network prediction. Characterization of the substrate usage capacity of the *R*. *solanacearum* strain GMI1000 and validation of the prediction capacity of the genome-scale metabolic model iRP1476.
(XLSX)

S4 Table Growth kinetics data. Experimental data of cultivation kinetics used for metabolic fluxes analysis.
(XLSX)

S5 Table Phenotypes microarray of *R*. *solanacearum* GMI1525, GMI1605, GMI1755. Characterization of the substrate usage capacity of the *R*. *solanacearum* deletion mutants GMI1525, GMI1605 and GMI1755.
(XLSX)

S6 Table List of substrates not used by the wild-type strain but used by the *phcA* mutant. (XLSX)
