# Supplementary material for: A Resource Allocation Trade-Off between Virulence and Proliferation Drives Metabolic Versatility in the Plant Pathogen Ralstonia solanacearum
Source: PLoS Pathog. 2016 Oct 12;12(10):e1005939. doi: 10.1371/journal.ppat.1005939 (PMC5061431; doi:10.1371/journal.ppat.1005939)
Supplement: S1 Fig — Phenotype Microarray data of strain GMI1000 on Biolog plates PM1 to PM10 incubated during 96h at a temperature of 28°C. The number of replicates is from 3 to 7. Data were treated with the R package opm. (PDF) [file ppat.1005939.s001.pdf]

Ralstonia solanacearum GMI1000 − PM01 Carbon source

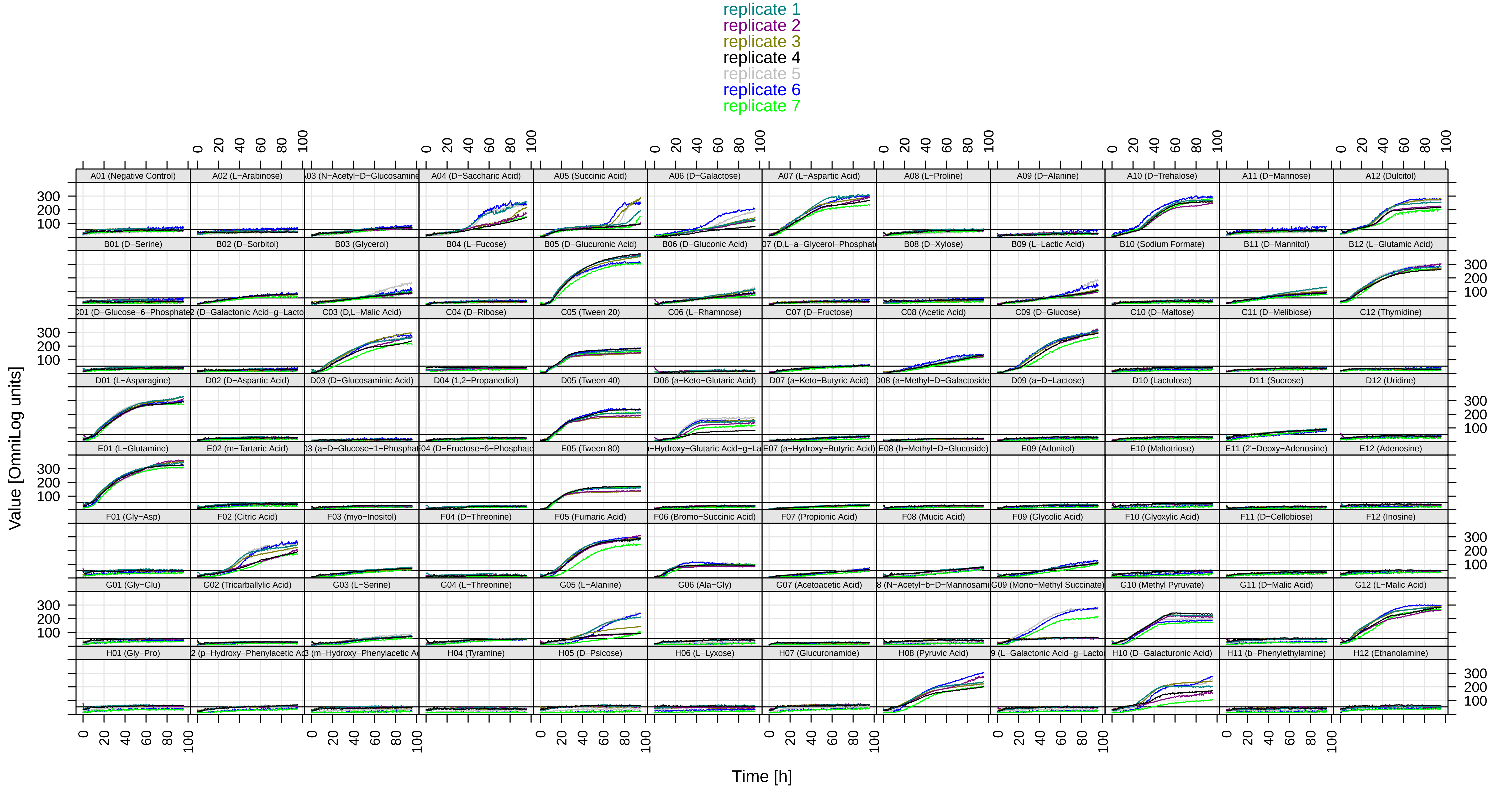

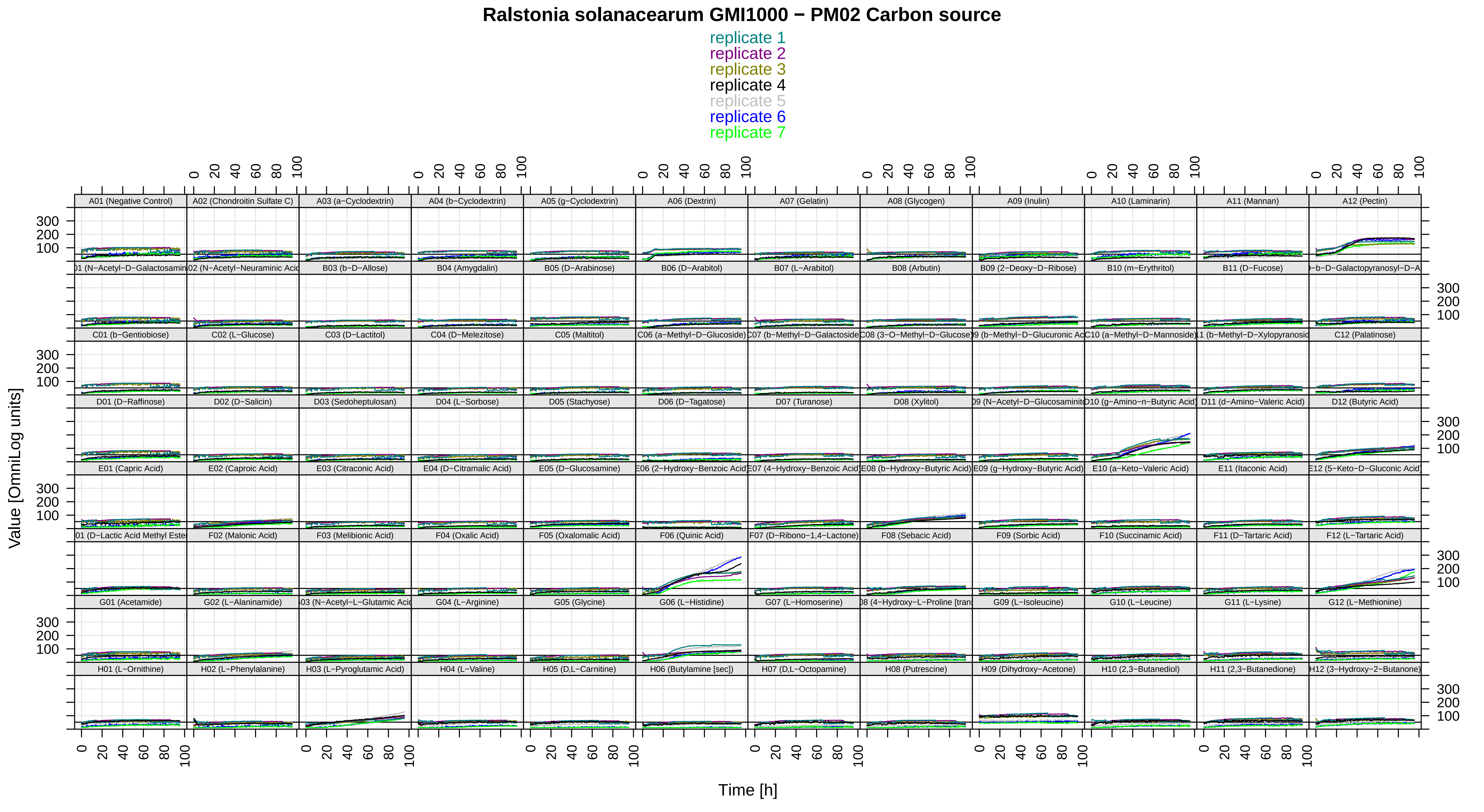

Ralstonia solanacearum GMI1000 − PM02 Carbon source
replicate 1
replicate 2
replicate 3
replicate 4
replicate 5
replicate 6
replicate 7
Value [OmniLog units]
Time [h]
A01 (Negative Control)
A02 (Chondroitin Sulfate C)
A03 (a−Cyclodextrin)
A04 (b−Cyclodextrin)
A05 (g−Cyclodextrin)
A06 (Dextrin)
A07 (Gelatin)
A08 (Glycogen)
A09 (Inulin)
A10 (Laminarin)
A11 (Mannan)
A12 (Pectin)
B03 (b−D−Allose)
B04 (Amygdalin)
B05 (D−Arabinose)
B06 (D−Arabitol)
B07 (L−Arabitol)
B08 (Arbutin)
B09 (2−Deoxy−D−Ribose)
B10 (m−Erythritol)
B11 (D−Fucose)
C01 (b−Gentiobiose)
C02 (L−Glucose)
C03 (D−Lactitol)
C04 (D−Melezitose)
C05 (Maltitol)
C06 (a−Methyl−D−Glucoside)
C12 (Palatinose)
D01 (D−Raffinose)
D02 (D−Salicin)
D03 (Sedoheptulosan)
D04 (L−Sorbose)
D05 (Stachyose)
D06 (D−Tagatose)
D07 (Turanose)
D08 (Xylitol)
D11 (d−Amino−Valeric Acid)
D12 (Butyric Acid)
E01 (Capric Acid)
E02 (Caproic Acid)
E03 (Citraconic Acid)
E04 (D−Citramalic Acid)
E05 (D−Glucosamine)
E08 (b−Hydroxy−Butyric Acid)
E09 (g−Hydroxy−Butyric Acid)
E10 (a−Keto−Valeric Acid)
E11 (Itaconic Acid)
F02 (Malonic Acid)
F03 (Melibionic Acid)
F04 (Oxalic Acid)
F05 (Oxalomalic Acid)
F06 (Quinic Acid)
F07 (D−Ribono−1,4−Lactone)
F08 (Sebacic Acid)
F09 (Sorbic Acid)
F10 (Succinamic Acid)
F11 (D−Tartaric Acid)
F12 (L−Tartaric Acid)
G01 (Acetamide)
G02 (L−Alaninamide)
G04 (L−Arginine)
G05 (Glycine)
G06 (L−Histidine)
G07 (L−Homoserine)
G09 (L−Isoleucine)
G10 (L−Leucine)
G11 (L−Lysine)
G12 (L−Methionine)
H01 (L−Ornithine)
H02 (L−Phenylalanine)
H03 (L−Pyroglutamic Acid)
H04 (L−Valine)
H05 (D,L−Carnitine)
H06 (Butylamine [sec])
H07 (D,L−Octopamine)
H08 (Putrescine)
H09 (Dihydroxy−Acetone)
H10 (2,3−Butanediol)
H11 (2,3−Butanedione)
H12 (3−Hydroxy−2−Butanone)

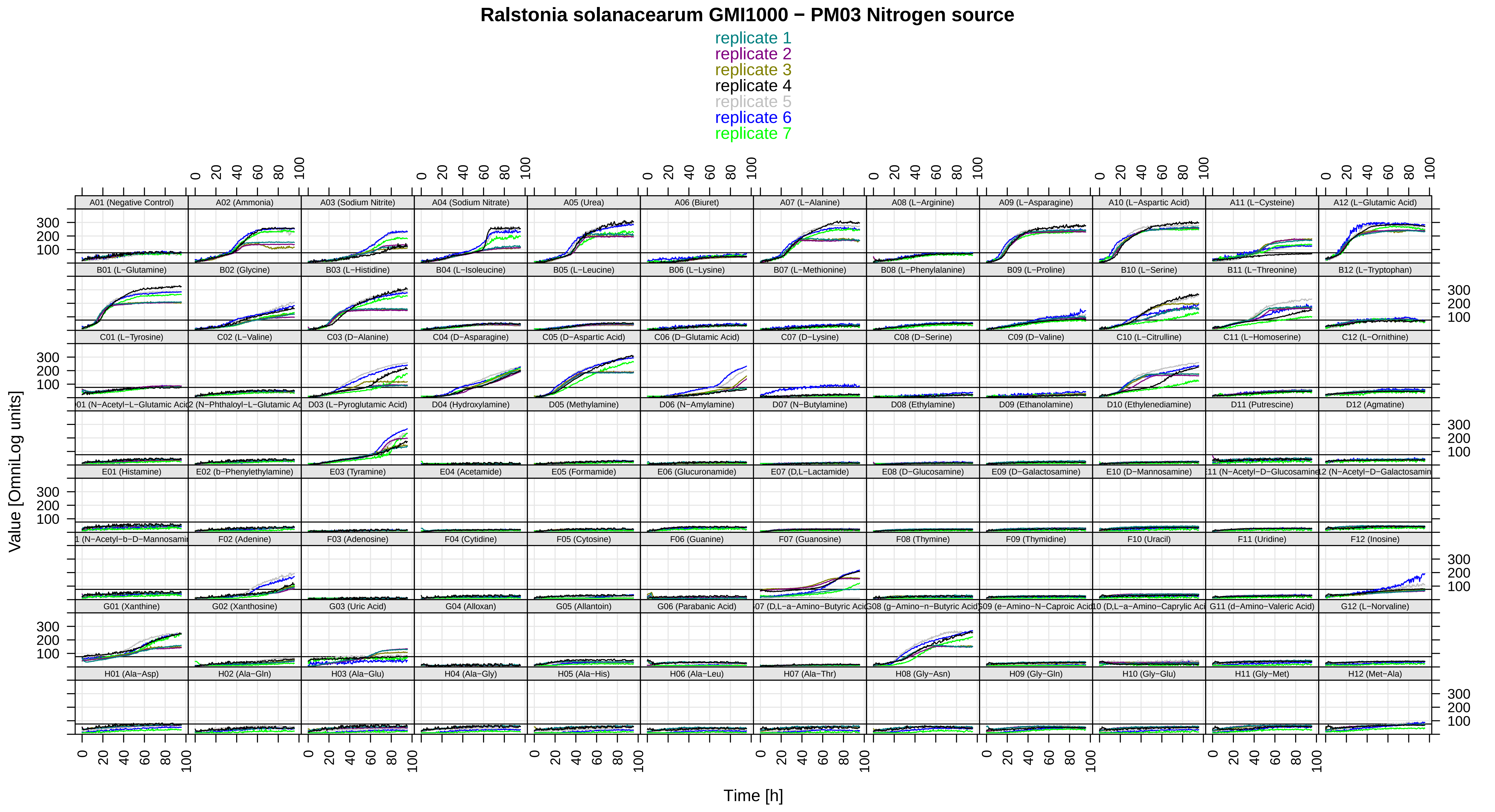

Ralstonia solanacearum GMI1000 − PM03 Nitrogen source
replicate 1
replicate 2
replicate 3
replicate 4
replicate 5
replicate 6
replicate 7
A01 (Negative Control)
A02 (Ammonia)
A03 (Sodium Nitrite)
A04 (Sodium Nitrate)
A05 (Urea)
A06 (Biuret)
A07 (L−Alanine)
A08 (L−Arginine)
A09 (L−Asparagine)
A10 (L−Aspartic Acid)
A11 (L−Cysteine)
A12 (L−Glutamic Acid)
B01 (L−Glutamine)
B02 (Glycine)
B03 (L−Histidine)
B04 (L−Isoleucine)
B05 (L−Leucine)
B06 (L−Lysine)
B07 (L−Methionine)
B08 (L−Phenylalanine)
B09 (L−Proline)
B10 (L−Serine)
B11 (L−Threonine)
B12 (L−Tryptophan)
C01 (L−Tyrosine)
C02 (L−Valine)
C03 (D−Alanine)
C04 (D−Asparagine)
C05 (D−Aspartic Acid)
C06 (D−Glutamic Acid)
C07 (D−Lysine)
C08 (D−Serine)
C09 (D−Valine)
C10 (L−Citrulline)
C11 (L−Homoserine)
C12 (L−Ornithine)
01 (N−Acetyl−L−Glutamic Acid
2 (N−Phthaloyl−L−Glutamic Ac
D03 (L−Pyroglutamic Acid)
D04 (Hydroxylamine)
D05 (Methylamine)
D06 (N−Amylamine)
D07 (N−Butylamine)
D08 (Ethylamine)
D09 (Ethanolamine)
D10 (Ethylenediamine)
D11 (Putrescine)
D12 (Agmatine)
E01 (Histamine)
E02 (b−Phenylethylamine)
E03 (Tyramine)
E04 (Acetamide)
E05 (Formamide)
E06 (Glucuronamide)
E07 (D,L−Lactamide)
E08 (D−Glucosamine)
E09 (D−Galactosamine)
E10 (D−Mannosamine)
11 (N−Acetyl−D−Glucosamine
2 (N−Acetyl−D−Galactosamin
1 (N−Acetyl−b−D−Mannosamin
F02 (Adenine)
F03 (Adenosine)
F04 (Cytidine)
F05 (Cytosine)
F06 (Guanine)
F07 (Guanosine)
F08 (Thymine)
F09 (Thymidine)
F10 (Uracil)
F11 (Uridine)
F12 (Inosine)
G01 (Xanthine)
G02 (Xanthosine)
G03 (Uric Acid)
G04 (Alloxan)
G05 (Allantoin)
G06 (Parabanic Acid)
07 (D,L−a−Amino−Butyric Acid
G08 (g−Amino−n−Butyric Acid)
09 (e−Amino−N−Caproic Acid
10 (D,L−a−Amino−Caprylic Aci
G11 (d−Amino−Valeric Acid)
G12 (L−Norvaline)
H01 (Ala−Asp)
H02 (Ala−Gln)
H03 (Ala−Glu)
H04 (Ala−Gly)
H05 (Ala−His)
H06 (Ala−Leu)
H07 (Ala−Thr)
H08 (Gly−Asn)
H09 (Gly−Gln)
H10 (Gly−Glu)
H11 (Gly−Met)
H12 (Met−Ala)
0 20 40 60 80 100
100 200 300
Value [OmniLog units]
Time [h]

Ralstonia solanacearum GMI1000 − PM04 Phosphorus source

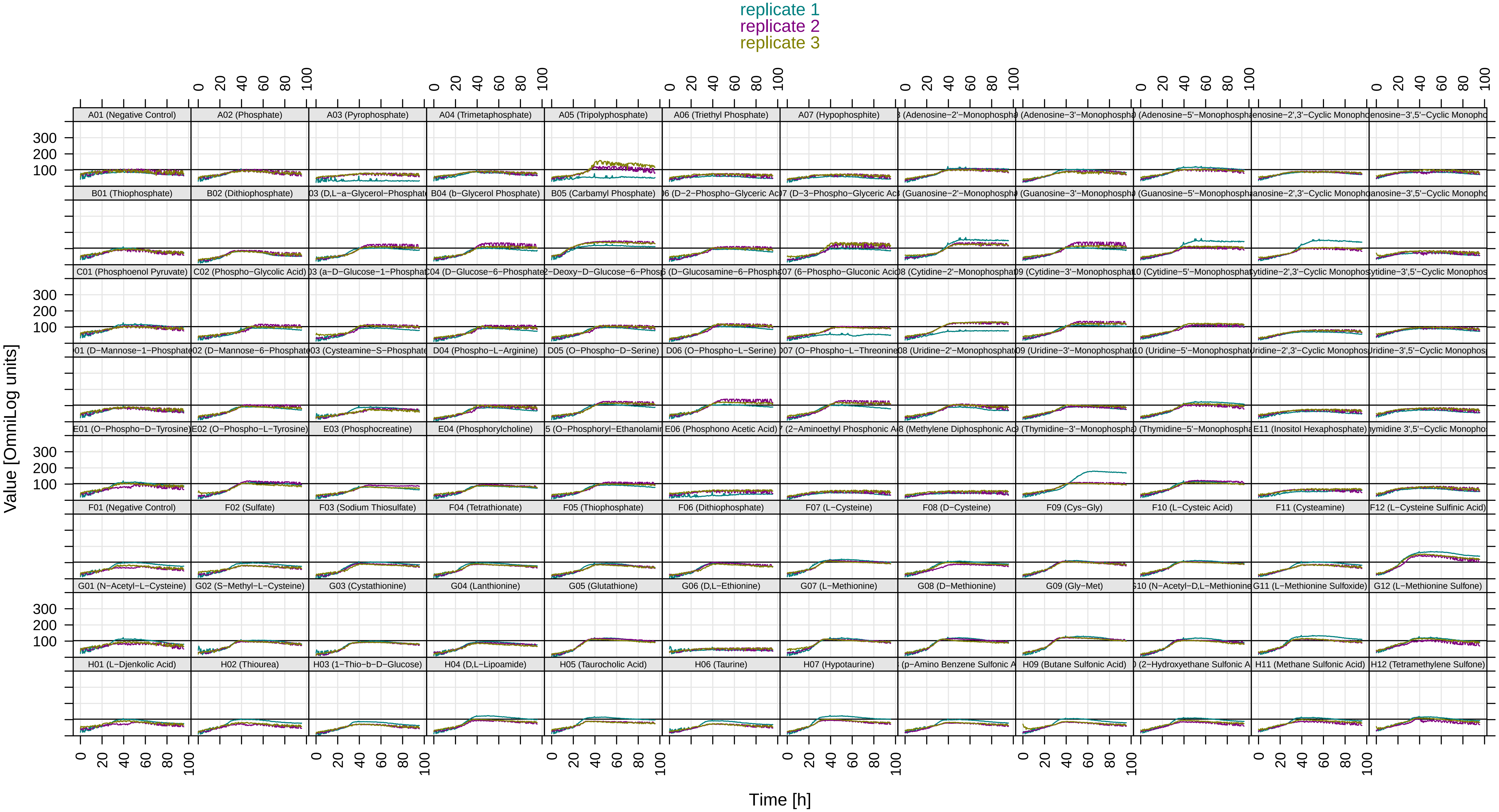

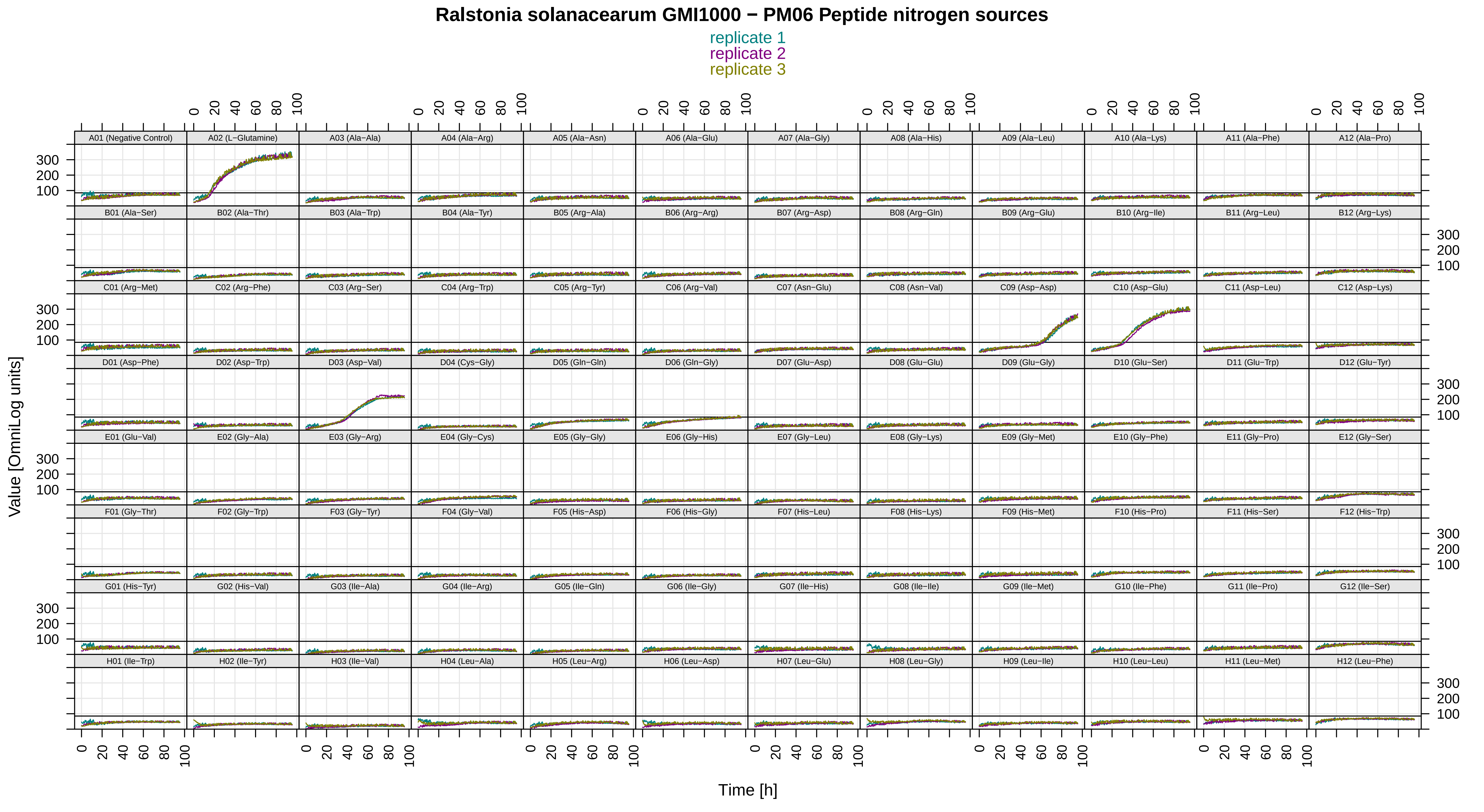
Ralstonia solanacearum GMI1000 − PM06 Peptide nitrogen sources
replicate 1
replicate 2
replicate 3
A01 (Negative Control)
A02 (L−Glutamine)
A03 (Ala−Ala)
A04 (Ala−Arg)
A05 (Ala−Asn)
A06 (Ala−Glu)
A07 (Ala−Gly)
A08 (Ala−His)
A09 (Ala−Leu)
A10 (Ala−Lys)
A11 (Ala−Phe)
A12 (Ala−Pro)
B01 (Ala−Ser)
B02 (Ala−Thr)
B03 (Ala−Trp)
B04 (Ala−Tyr)
B05 (Arg−Ala)
B06 (Arg−Arg)
B07 (Arg−Asp)
B08 (Arg−Gln)
B09 (Arg−Glu)
B10 (Arg−Ile)
B11 (Arg−Leu)
B12 (Arg−Lys)
C01 (Arg−Met)
C02 (Arg−Phe)
C03 (Arg−Ser)
C04 (Arg−Trp)
C05 (Arg−Tyr)
C06 (Arg−Val)
C07 (Asn−Glu)
C08 (Asn−Val)
C09 (Asp−Asp)
C10 (Asp−Glu)
C11 (Asp−Leu)
C12 (Asp−Lys)
D01 (Asp−Phe)
D02 (Asp−Trp)
D03 (Asp−Val)
D04 (Cys−Gly)
D05 (Gln−Gln)
D06 (Gln−Gly)
D07 (Glu−Asp)
D08 (Glu−Glu)
D09 (Glu−Gly)
D10 (Glu−Ser)
D11 (Glu−Trp)
D12 (Glu−Tyr)
E01 (Glu−Val)
E02 (Gly−Ala)
E03 (Gly−Arg)
E04 (Gly−Cys)
E05 (Gly−Gly)
E06 (Gly−His)
E07 (Gly−Leu)
E08 (Gly−Lys)
E09 (Gly−Met)
E10 (Gly−Phe)
E11 (Gly−Pro)
E12 (Gly−Ser)
F01 (Gly−Thr)
F02 (Gly−Trp)
F03 (Gly−Tyr)
F04 (Gly−Val)
F05 (His−Asp)
F06 (His−Gly)
F07 (His−Leu)
F08 (His−Lys)
F09 (His−Met)
F10 (His−Pro)
F11 (His−Ser)
F12 (His−Trp)
G01 (His−Tyr)
G02 (His−Val)
G03 (Ile−Ala)
G04 (Ile−Arg)
G05 (Ile−Gln)
G06 (Ile−Gly)
G07 (Ile−His)
G08 (Ile−Ile)
G09 (Ile−Met)
G10 (Ile−Phe)
G11 (Ile−Pro)
G12 (Ile−Ser)
H01 (Ile−Trp)
H02 (Ile−Tyr)
H03 (Ile−Val)
H04 (Leu−Ala)
H05 (Leu−Arg)
H06 (Leu−Asp)
H07 (Leu−Glu)
H08 (Leu−Gly)
H09 (Leu−Ile)
H10 (Leu−Leu)
H11 (Leu−Met)
H12 (Leu−Phe)
Value [OmniLog units]
Time [h]
0 20 40 60 80 100
100 200 300

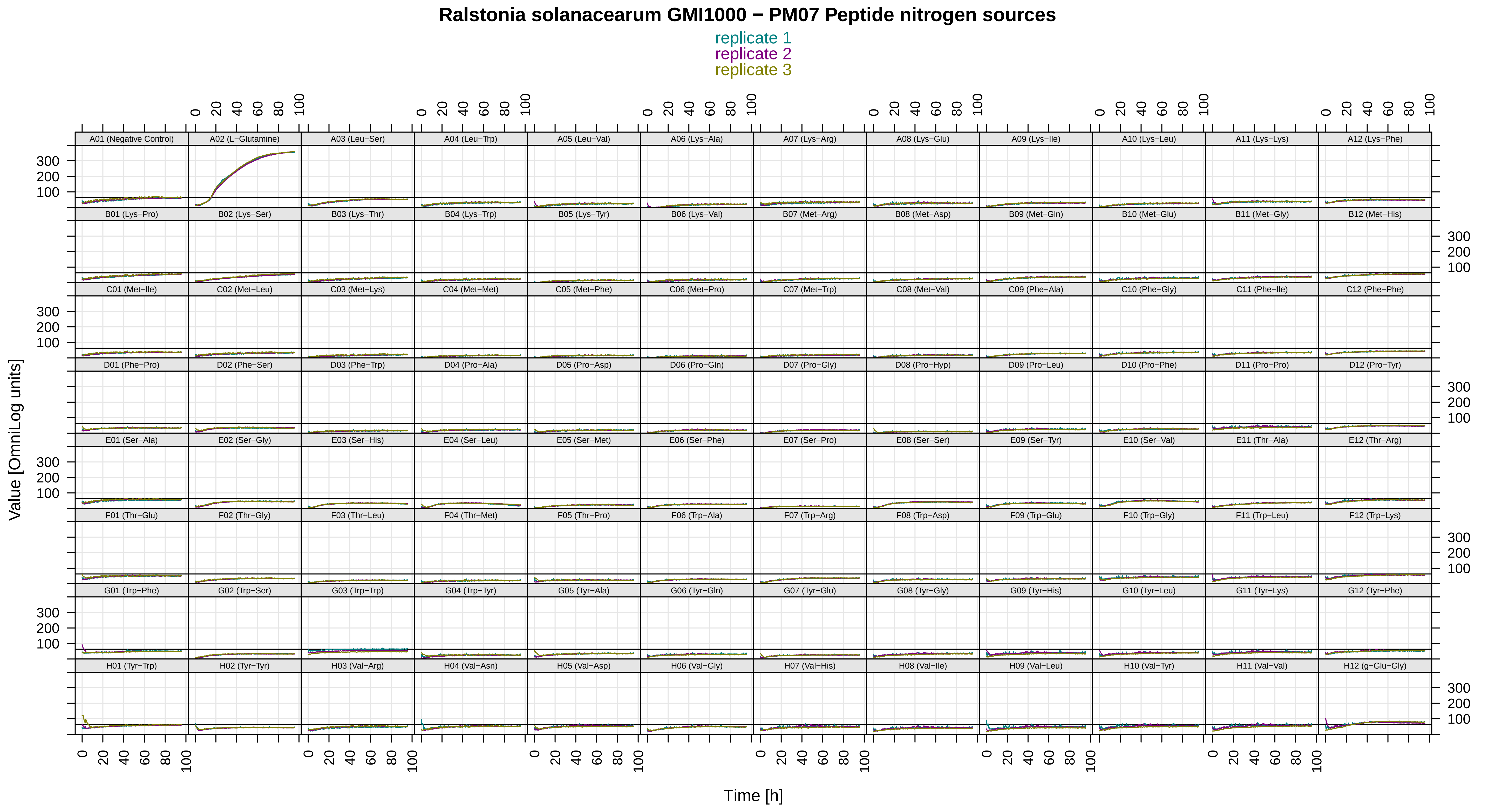
Ralstonia solanacearum GMI1000 − PM07 Peptide nitrogen sources
replicate 1
replicate 2
replicate 3
A01 (Negative Control)
A02 (L−Glutamine)
A03 (Leu−Ser)
A04 (Leu−Trp)
A05 (Leu−Val)
A06 (Lys−Ala)
A07 (Lys−Arg)
A08 (Lys−Glu)
A09 (Lys−Ile)
A10 (Lys−Leu)
A11 (Lys−Lys)
A12 (Lys−Phe)
B01 (Lys−Pro)
B02 (Lys−Ser)
B03 (Lys−Thr)
B04 (Lys−Trp)
B05 (Lys−Tyr)
B06 (Lys−Val)
B07 (Met−Arg)
B08 (Met−Asp)
B09 (Met−Gln)
B10 (Met−Glu)
B11 (Met−Gly)
B12 (Met−His)
C01 (Met−Ile)
C02 (Met−Leu)
C03 (Met−Lys)
C04 (Met−Met)
C05 (Met−Phe)
C06 (Met−Pro)
C07 (Met−Trp)
C08 (Met−Val)
C09 (Phe−Ala)
C10 (Phe−Gly)
C11 (Phe−Ile)
C12 (Phe−Phe)
D01 (Phe−Pro)
D02 (Phe−Ser)
D03 (Phe−Trp)
D04 (Pro−Ala)
D05 (Pro−Asp)
D06 (Pro−Gln)
D07 (Pro−Gly)
D08 (Pro−Hyp)
D09 (Pro−Leu)
D10 (Pro−Phe)
D11 (Pro−Pro)
D12 (Pro−Tyr)
E01 (Ser−Ala)
E02 (Ser−Gly)
E03 (Ser−His)
E04 (Ser−Leu)
E05 (Ser−Met)
E06 (Ser−Phe)
E07 (Ser−Pro)
E08 (Ser−Ser)
E09 (Ser−Tyr)
E10 (Ser−Val)
E11 (Thr−Ala)
E12 (Thr−Arg)
F01 (Thr−Glu)
F02 (Thr−Gly)
F03 (Thr−Leu)
F04 (Thr−Met)
F05 (Thr−Pro)
F06 (Trp−Ala)
F07 (Trp−Arg)
F08 (Trp−Asp)
F09 (Trp−Glu)
F10 (Trp−Gly)
F11 (Trp−Leu)
F12 (Trp−Lys)
G01 (Trp−Phe)
G02 (Trp−Ser)
G03 (Trp−Trp)
G04 (Trp−Tyr)
G05 (Tyr−Ala)
G06 (Tyr−Gln)
G07 (Tyr−Glu)
G08 (Tyr−Gly)
G09 (Tyr−His)
G10 (Tyr−Leu)
G11 (Tyr−Lys)
G12 (Tyr−Phe)
H01 (Tyr−Trp)
H02 (Tyr−Tyr)
H03 (Val−Arg)
H04 (Val−Asn)
H05 (Val−Asp)
H06 (Val−Gly)
H07 (Val−His)
H08 (Val−Ile)
H09 (Val−Leu)
H10 (Val−Tyr)
H11 (Val−Val)
H12 (g−Glu−Gly)
Value [OmniLog units]
Time [h]
0
20
40
60
80
100
200
300

Ralstonia solanacearum GMI1000 − PM08 Peptide nitrogen sources

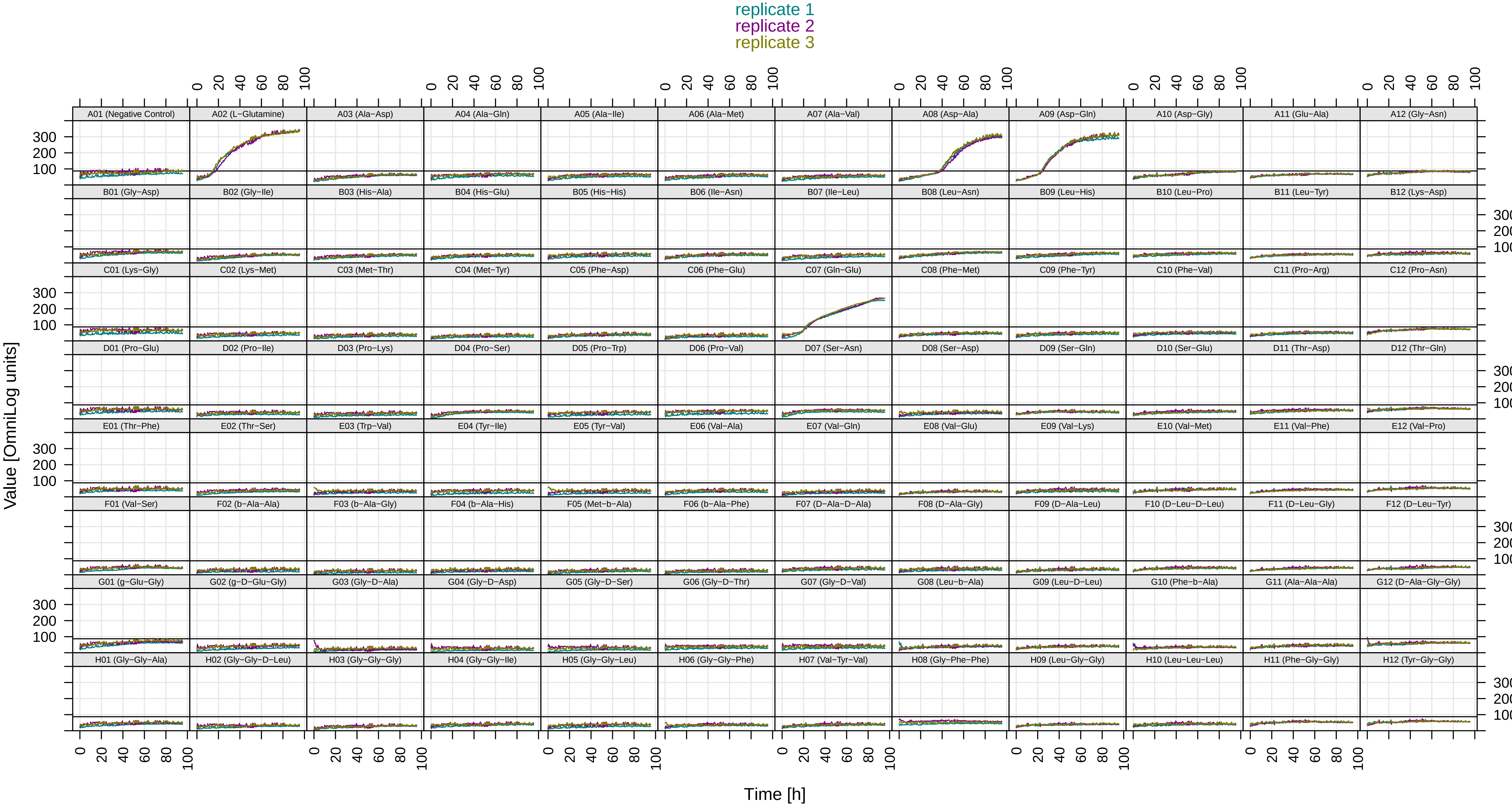

Ralstonia solanacearum GMI1000 − PM09 Osmolytes

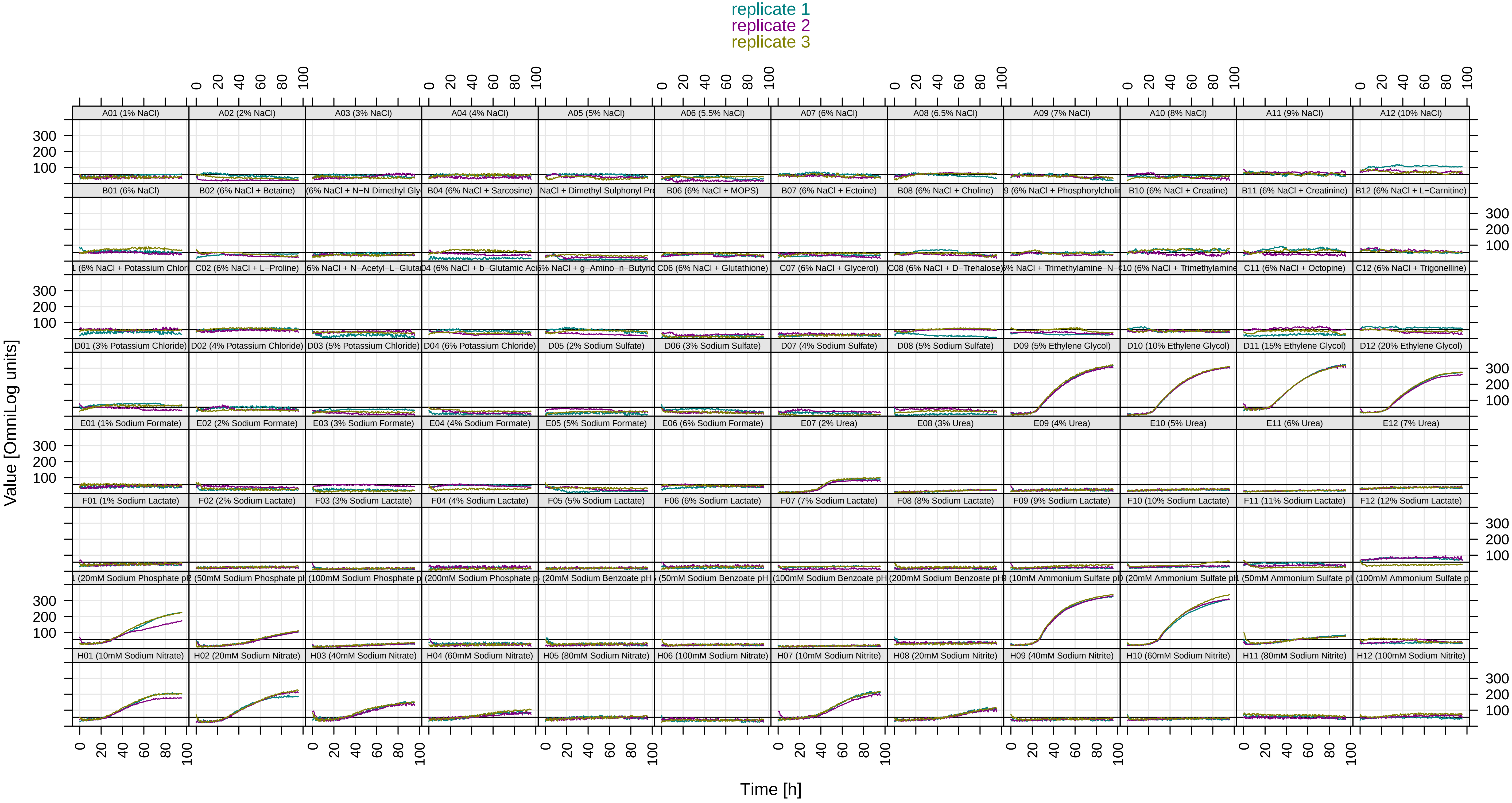

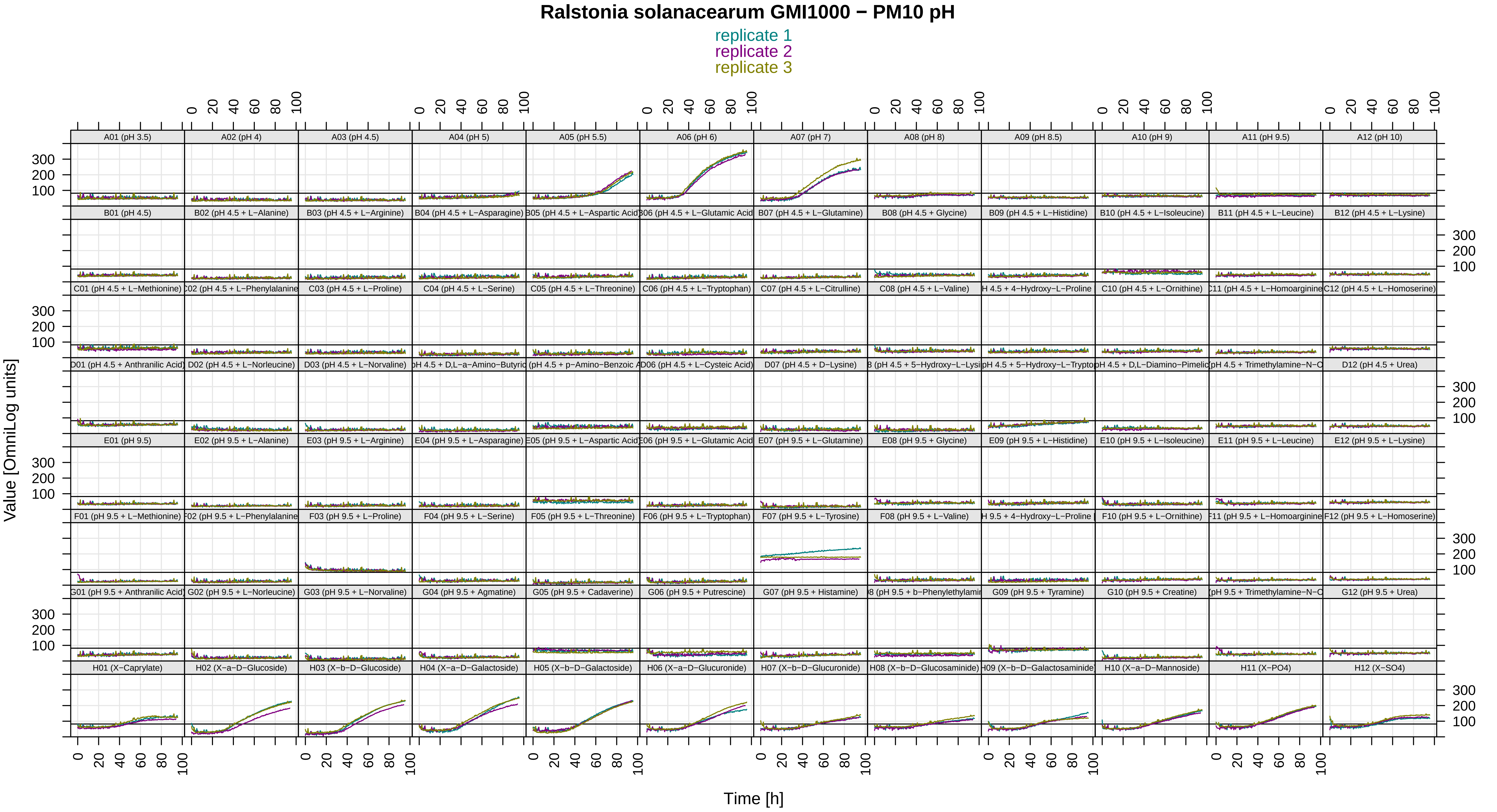
Ralstonia solanacearum GMI1000 − PM10 pH
replicate 1
replicate 2
replicate 3
Value [OmniLog units]
Time [h]
A01 (pH 3.5)
A02 (pH 4)
A03 (pH 4.5)
A04 (pH 5)
A05 (pH 5.5)
A06 (pH 6)
A07 (pH 7)
A08 (pH 8)
A09 (pH 8.5)
A10 (pH 9)
A11 (pH 9.5)
A12 (pH 10)
B01 (pH 4.5)
B02 (pH 4.5 + L−Alanine)
B03 (pH 4.5 + L−Arginine)
B04 (pH 4.5 + L−Asparagine)
B08 (pH 4.5 + Glycine)
B09 (pH 4.5 + L−Histidine)
B10 (pH 4.5 + L−Isoleucine)
B11 (pH 4.5 + L−Leucine)
B12 (pH 4.5 + L−Lysine)
C01 (pH 4.5 + L−Methionine)
C03 (pH 4.5 + L−Proline)
C04 (pH 4.5 + L−Serine)
C05 (pH 4.5 + L−Threonine)
C06 (pH 4.5 + L−Tryptophan)
C07 (pH 4.5 + L−Citrulline)
C08 (pH 4.5 + L−Valine)
C10 (pH 4.5 + L−Ornithine)
C12 (pH 4.5 + L−Homoserine)
D01 (pH 4.5 + Anthranilic Acid)
D02 (pH 4.5 + L−Norleucine)
D03 (pH 4.5 + L−Norvaline)
D06 (pH 4.5 + L−Cysteic Acid)
D07 (pH 4.5 + D−Lysine)
D12 (pH 4.5 + Urea)
E01 (pH 9.5)
E02 (pH 9.5 + L−Alanine)
E03 (pH 9.5 + L−Arginine)
E04 (pH 9.5 + L−Asparagine)
E07 (pH 9.5 + L−Glutamine)
E08 (pH 9.5 + Glycine)
E09 (pH 9.5 + L−Histidine)
E10 (pH 9.5 + L−Isoleucine)
E11 (pH 9.5 + L−Leucine)
E12 (pH 9.5 + L−Lysine)
F01 (pH 9.5 + L−Methionine)
F03 (pH 9.5 + L−Proline)
F04 (pH 9.5 + L−Serine)
F05 (pH 9.5 + L−Threonine)
F06 (pH 9.5 + L−Tryptophan)
F07 (pH 9.5 + L−Tyrosine)
F08 (pH 9.5 + L−Valine)
F10 (pH 9.5 + L−Ornithine)
F12 (pH 9.5 + L−Homoserine)
G02 (pH 9.5 + L−Norleucine)
G03 (pH 9.5 + L−Norvaline)
G04 (pH 9.5 + Agmatine)
G05 (pH 9.5 + Cadaverine)
G06 (pH 9.5 + Putrescine)
G07 (pH 9.5 + Histamine)
G09 (pH 9.5 + Tyramine)
G10 (pH 9.5 + Creatine)
G12 (pH 9.5 + Urea)
H01 (X−Caprylate)
H02 (X−a−D−Glucoside)
H03 (X−b−D−Glucoside)
H04 (X−a−D−Galactoside)
H05 (X−b−D−Galactoside)
H06 (X−a−D−Glucuronide)
H07 (X−b−D−Glucuronide)
H08 (X−b−D−Glucosaminide)
H10 (X−a−D−Mannoside)
H11 (X−PO4)
H12 (X−SO4)
