# Supplementary figures and images for: A Resource Allocation Trade-Off between Virulence and Proliferation Drives Metabolic Versatility in the Plant Pathogen Ralstonia solanacearum
Source: PLoS Pathog. 2016 Oct 12;12(10):e1005939. doi: 10.1371/journal.ppat.1005939 (PMC5061431; doi:10.1371/journal.ppat.1005939)

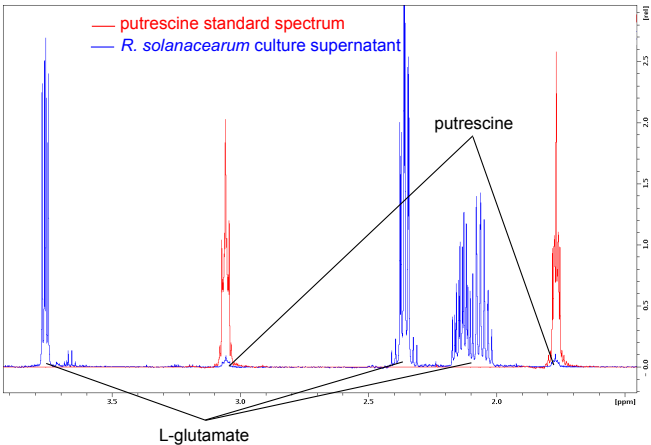

Supplement: S2 Fig — (PDF) [file ppat.1005939.s002.pdf]

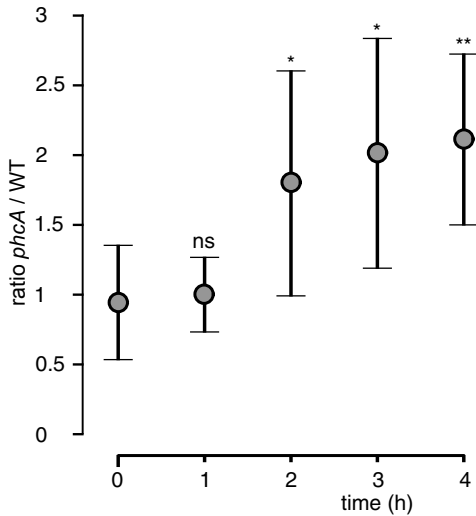

Supplement: S3 Fig — Liquid cultures were inoculated with an equal proportion of the GMI1000 and the phcA mutant, 5·107 cell.ml-1. Significance level: ns, not significant; *, < 0.05; **, <0.01. (PDF) [file ppat.1005939.s003.pdf]

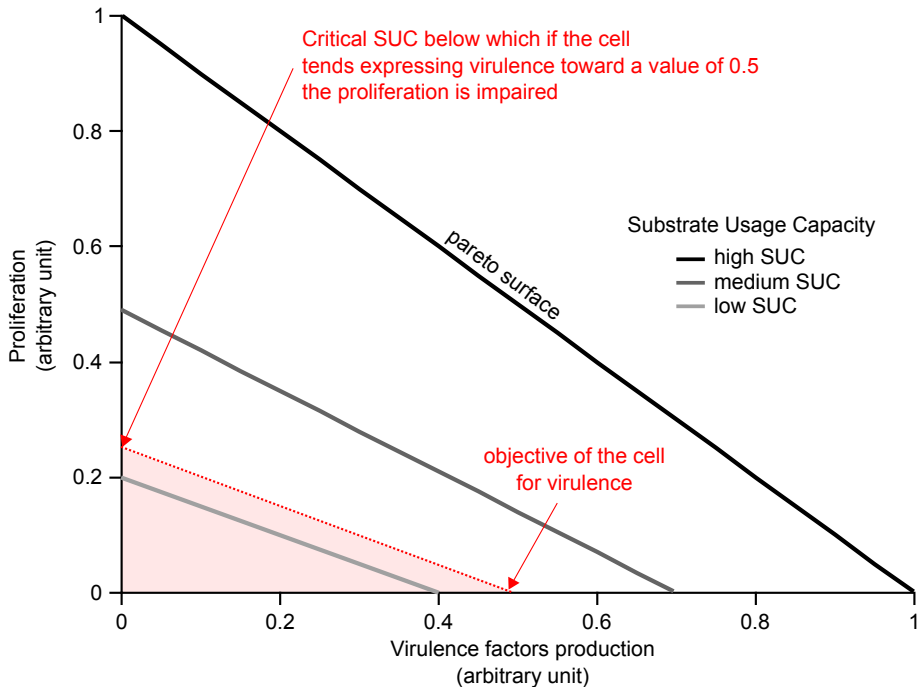

Supplement: S4 Fig — For each SUC considered the pareto surface, which corresponds to the trade-off surface optimizing both objectives, is drawn. Medium and low SUC were obtained by decreasing substrate uptake rate as yield. (PDF) [file ppat.1005939.s004.pdf]

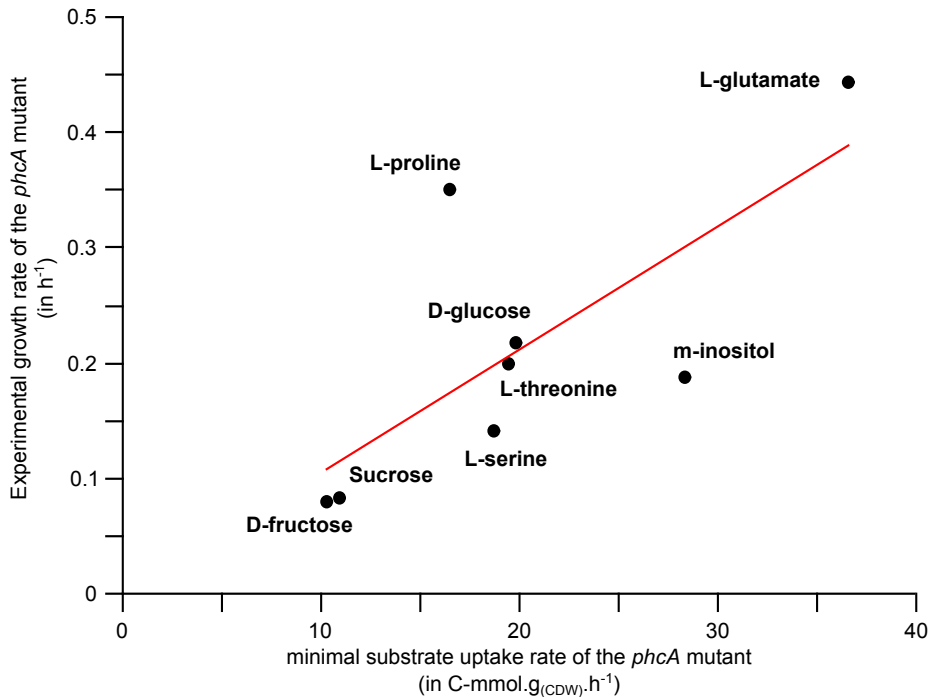

Supplement: S6 Fig — The linear correlation (red line) is 0.53. (PDF) [file ppat.1005939.s006.pdf]

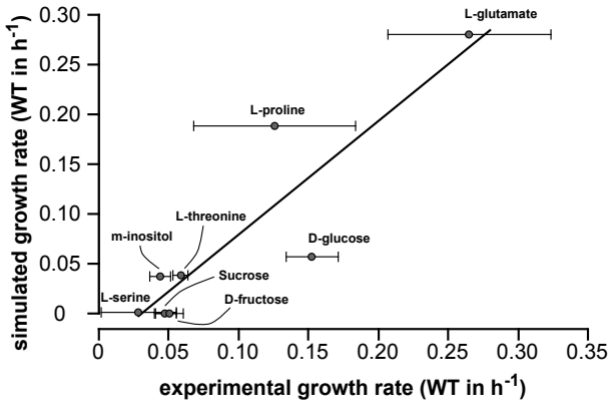

Supplement: S7 Fig — The linear correlation (black line) is 0.80. The simulated growth rates were calculated by FBA using the minimal substrate uptake rates of the phcA mutant plus the cost of virulence factors determined previously as constraints. (PDF) [file ppat.1005939.s007.pdf]
