# Supplementary material for: A Resource Allocation Trade-Off between Virulence and Proliferation Drives Metabolic Versatility in the Plant Pathogen Ralstonia solanacearum
Source: PLoS Pathog. 2016 Oct 12;12(10):e1005939. doi: 10.1371/journal.ppat.1005939 (PMC5061431; doi:10.1371/journal.ppat.1005939)
Supplement: S5 Fig — Phenotype Microarray data were collected upon 96h at a temperature of 28°C for the plates PM1, PM2 and PM3. Three replicates were performed. Data were treated with the R package opm. (PDF) [file ppat.1005939.s005.pdf]

Ralstonia solanacearum GMI1525 − PM01 Carbon source

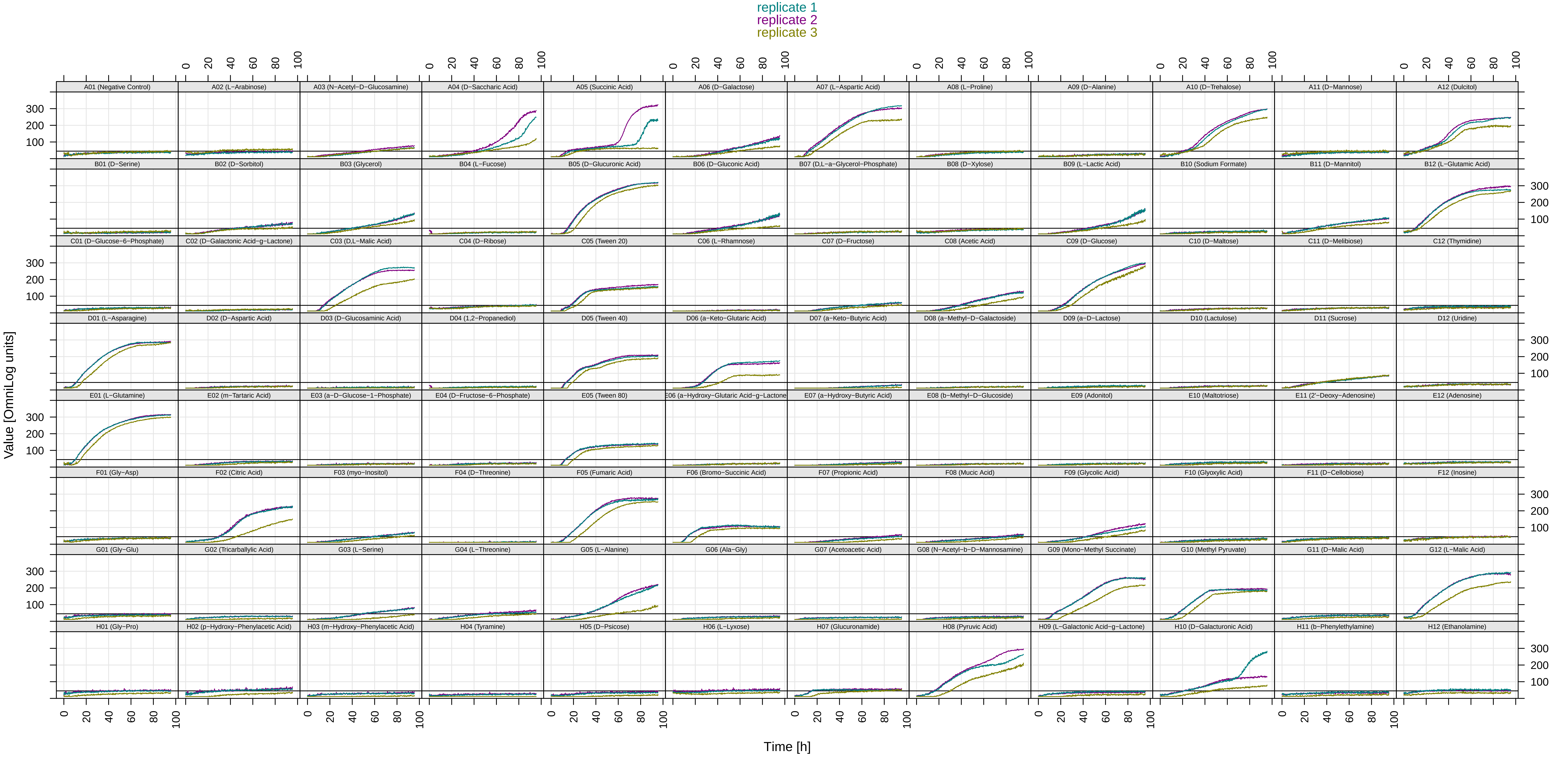

Ralstonia solanacearum GMI1525 − PM02 Carbon source

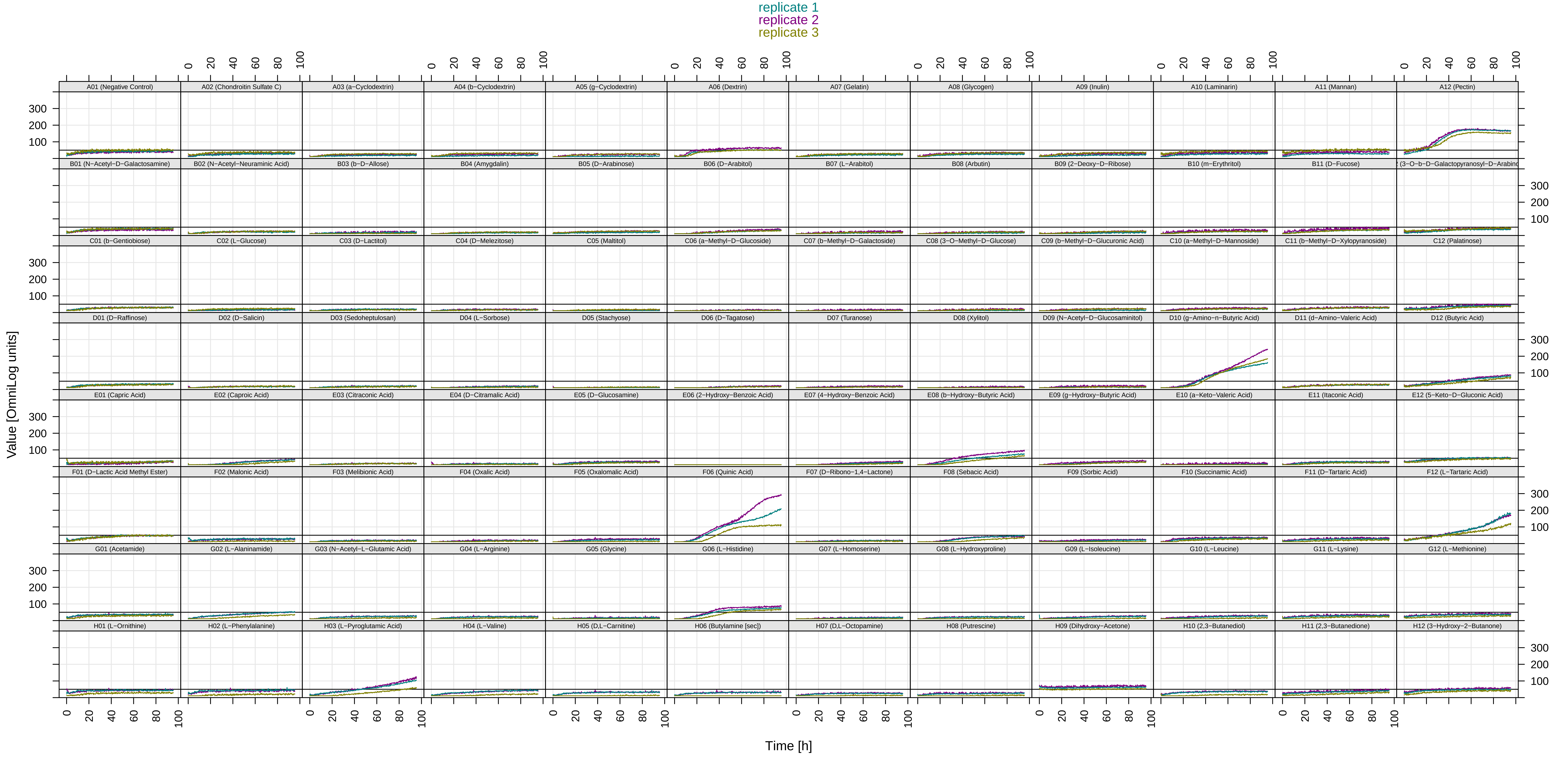

Ralstonia solanacearum GMI1525 − PM03 Nitrogen source

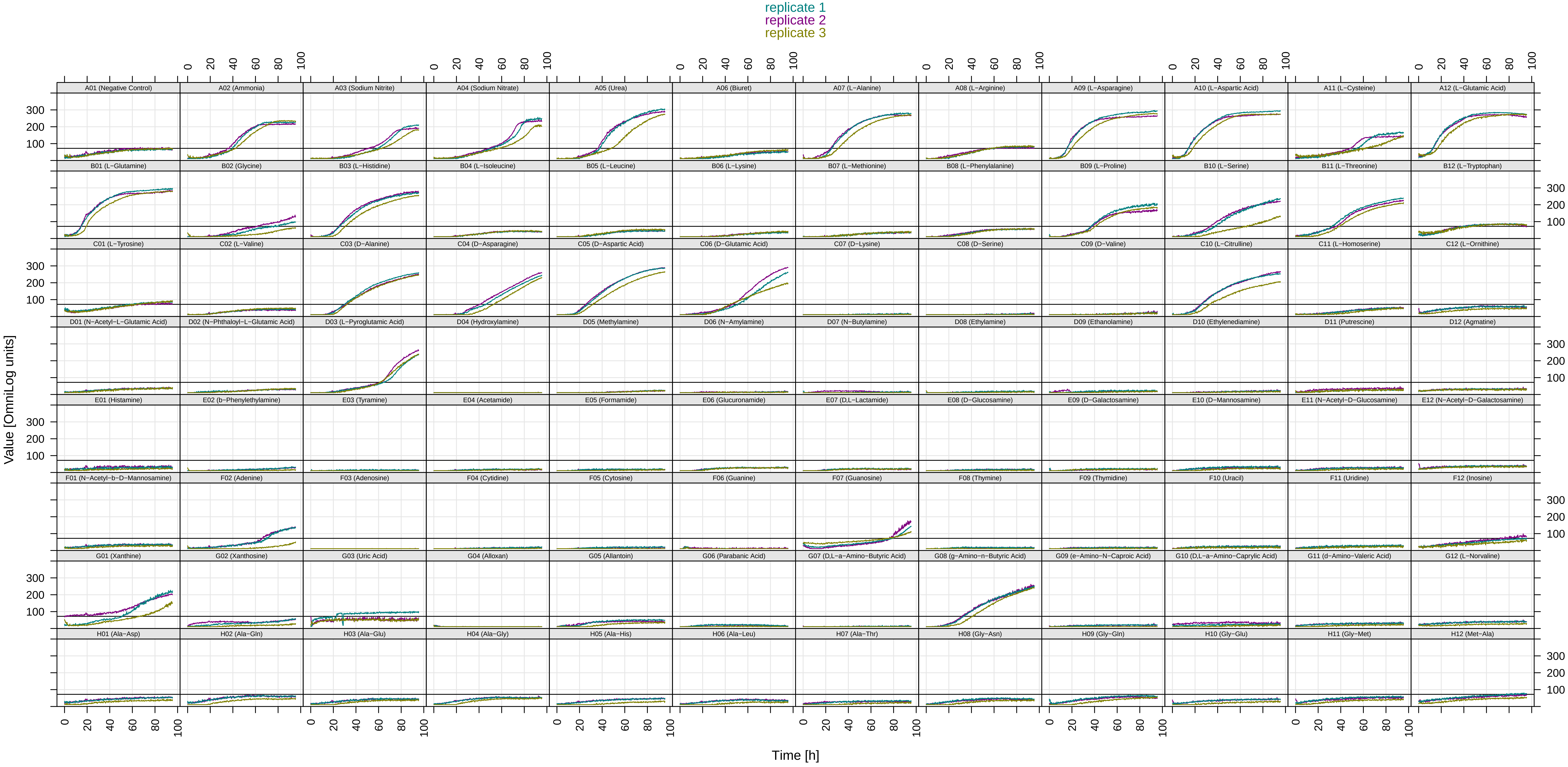

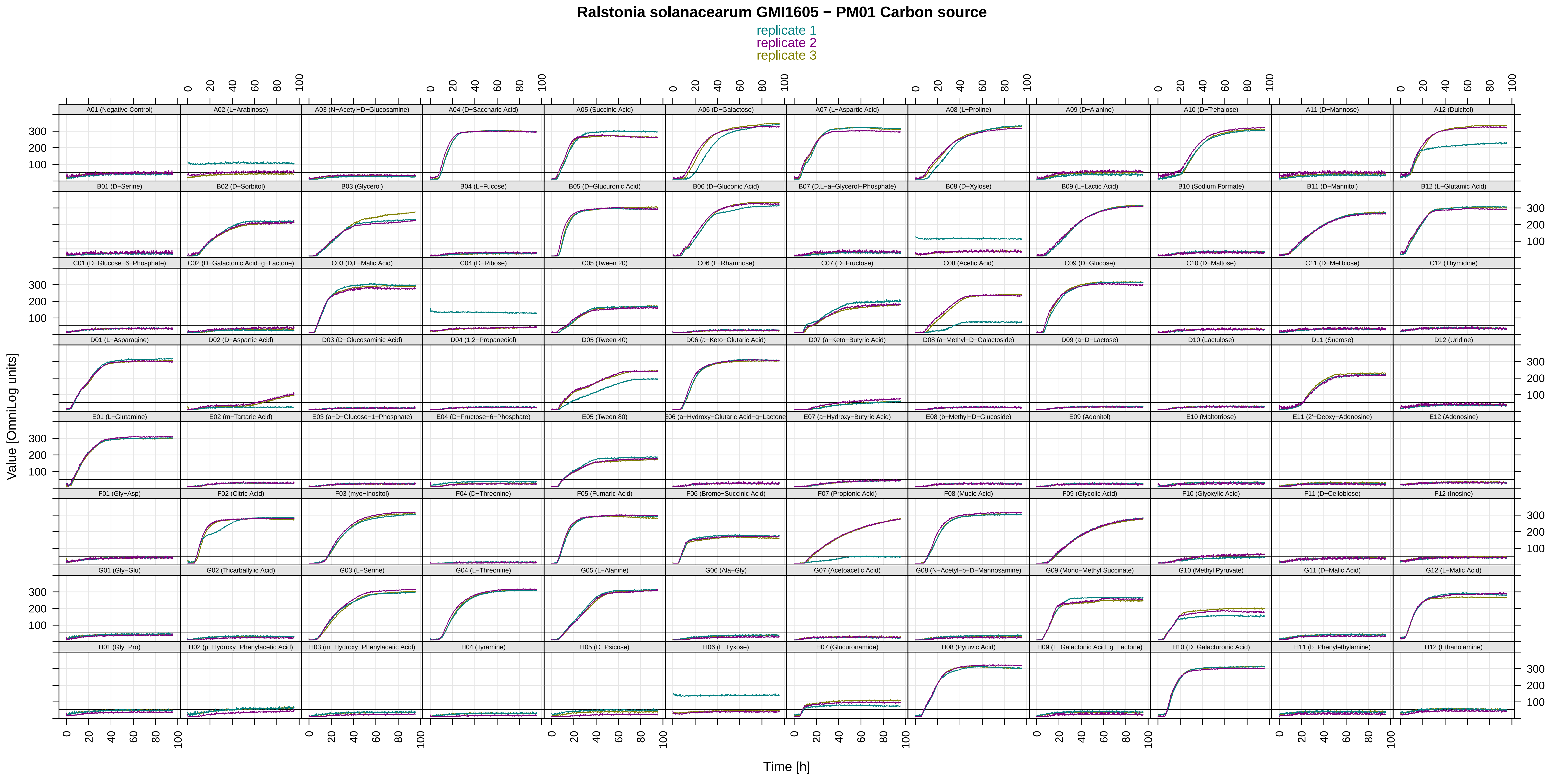

Ralstonia solanacearum GMI1605 − PM01 Carbon source
replicate 1
replicate 2
replicate 3
Value [OmniLog units]
Time [h]
A01 (Negative Control)
A02 (L−Arabinose)
A03 (N−Acetyl−D−Glucosamine)
A04 (D−Saccharic Acid)
A05 (Succinic Acid)
A06 (D−Galactose)
A07 (L−Aspartic Acid)
A08 (L−Proline)
A09 (D−Alanine)
A10 (D−Trehalose)
A11 (D−Mannose)
A12 (Dulcitol)
B01 (D−Serine)
B02 (D−Sorbitol)
B03 (Glycerol)
B04 (L−Fucose)
B05 (D−Glucuronic Acid)
B06 (D−Gluconic Acid)
B07 (D,L−a−Glycerol−Phosphate)
B08 (D−Xylose)
B09 (L−Lactic Acid)
B10 (Sodium Formate)
B11 (D−Mannitol)
B12 (L−Glutamic Acid)
C01 (D−Glucose−6−Phosphate)
C02 (D−Galactonic Acid−g−Lactone)
C03 (D,L−Malic Acid)
C04 (D−Ribose)
C05 (Tween 20)
C06 (L−Rhamnose)
C07 (D−Fructose)
C08 (Acetic Acid)
C09 (D−Glucose)
C10 (D−Maltose)
C11 (D−Melibiose)
C12 (Thymidine)
D01 (L−Asparagine)
D02 (D−Aspartic Acid)
D03 (D−Glucosaminic Acid)
D04 (1,2−Propanediol)
D05 (Tween 40)
D06 (a−Keto−Glutaric Acid)
D07 (a−Keto−Butyric Acid)
D08 (a−Methyl−D−Galactoside)
D09 (a−D−Lactose)
D10 (Lactulose)
D11 (Sucrose)
D12 (Uridine)
E01 (L−Glutamine)
E02 (m−Tartaric Acid)
E03 (a−D−Glucose−1−Phosphate)
E04 (D−Fructose−6−Phosphate)
E05 (Tween 80)
E06 (a−Hydroxy−Glutaric Acid−g−Lactone)
E07 (a−Hydroxy−Butyric Acid)
E08 (b−Methyl−D−Glucoside)
E09 (Adonitol)
E10 (Maltotriose)
E11 (2'−Deoxy−Adenosine)
E12 (Adenosine)
F01 (Gly−Asp)
F02 (Citric Acid)
F03 (myo−Inositol)
F04 (D−Threonine)
F05 (Fumaric Acid)
F06 (Bromo−Succinic Acid)
F07 (Propionic Acid)
F08 (Mucic Acid)
F09 (Glycolic Acid)
F10 (Glyoxylic Acid)
F11 (D−Cellobiose)
F12 (Inosine)
G01 (Gly−Glu)
G02 (Tricarballylic Acid)
G03 (L−Serine)
G04 (L−Threonine)
G05 (L−Alanine)
G06 (Ala−Gly)
G07 (Acetoacetic Acid)
G08 (N−Acetyl−b−D−Mannosamine)
G09 (Mono−Methyl Succinate)
G10 (Methyl Pyruvate)
G11 (D−Malic Acid)
G12 (L−Malic Acid)
H01 (Gly−Pro)
H02 (p−Hydroxy−Phenylacetic Acid)
H03 (m−Hydroxy−Phenylacetic Acid)
H04 (Tyramine)
H05 (D−Psicose)
H06 (L−Lyxose)
H07 (Glucuronamide)
H08 (Pyruvic Acid)
H09 (L−Galactonic Acid−g−Lactone)
H10 (D−Galacturonic Acid)
H11 (b−Phenylethylamine)
H12 (Ethanolamine)

Ralstonia solanacearum GMI1605 − PM02 Carbon source

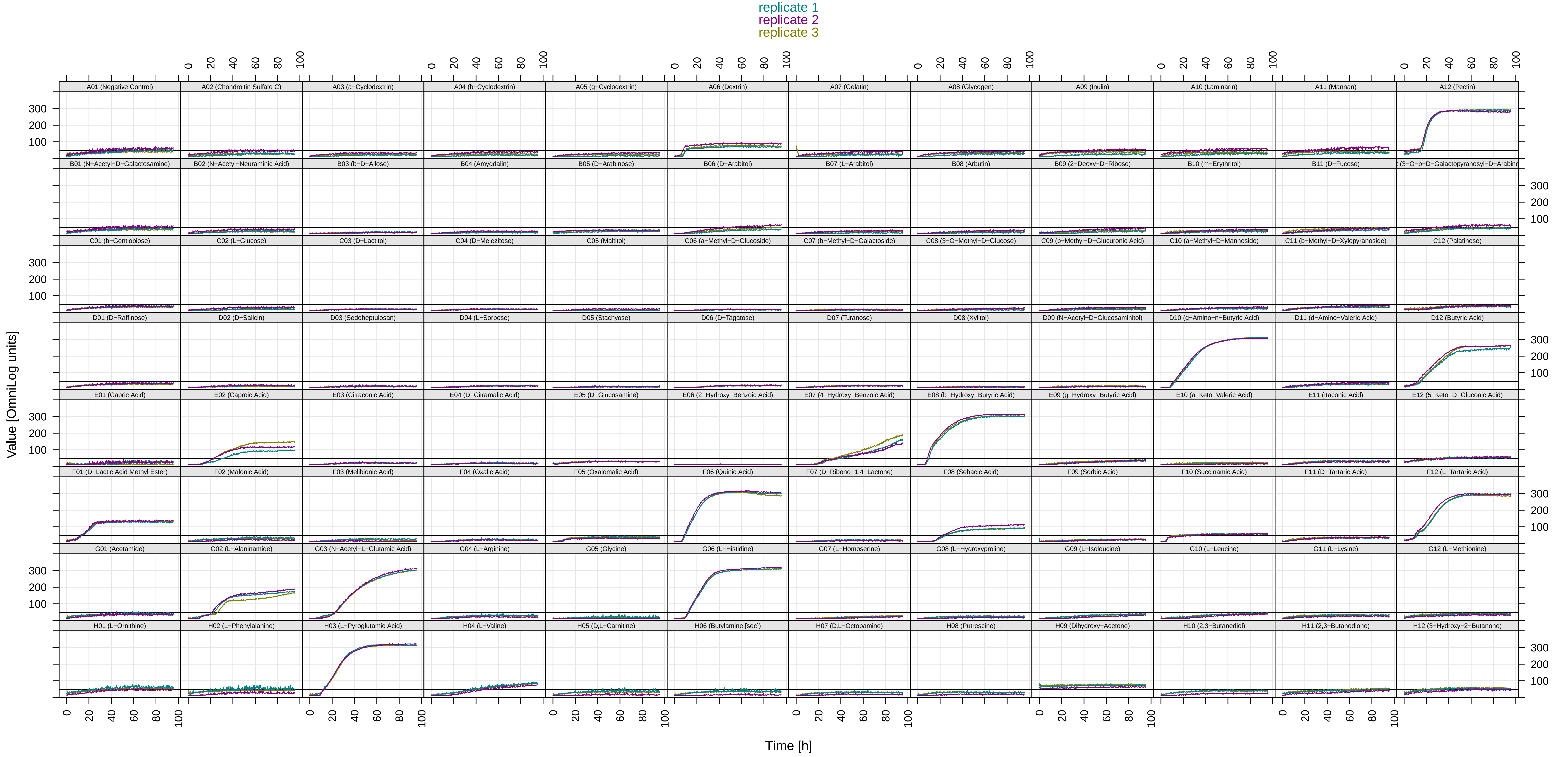

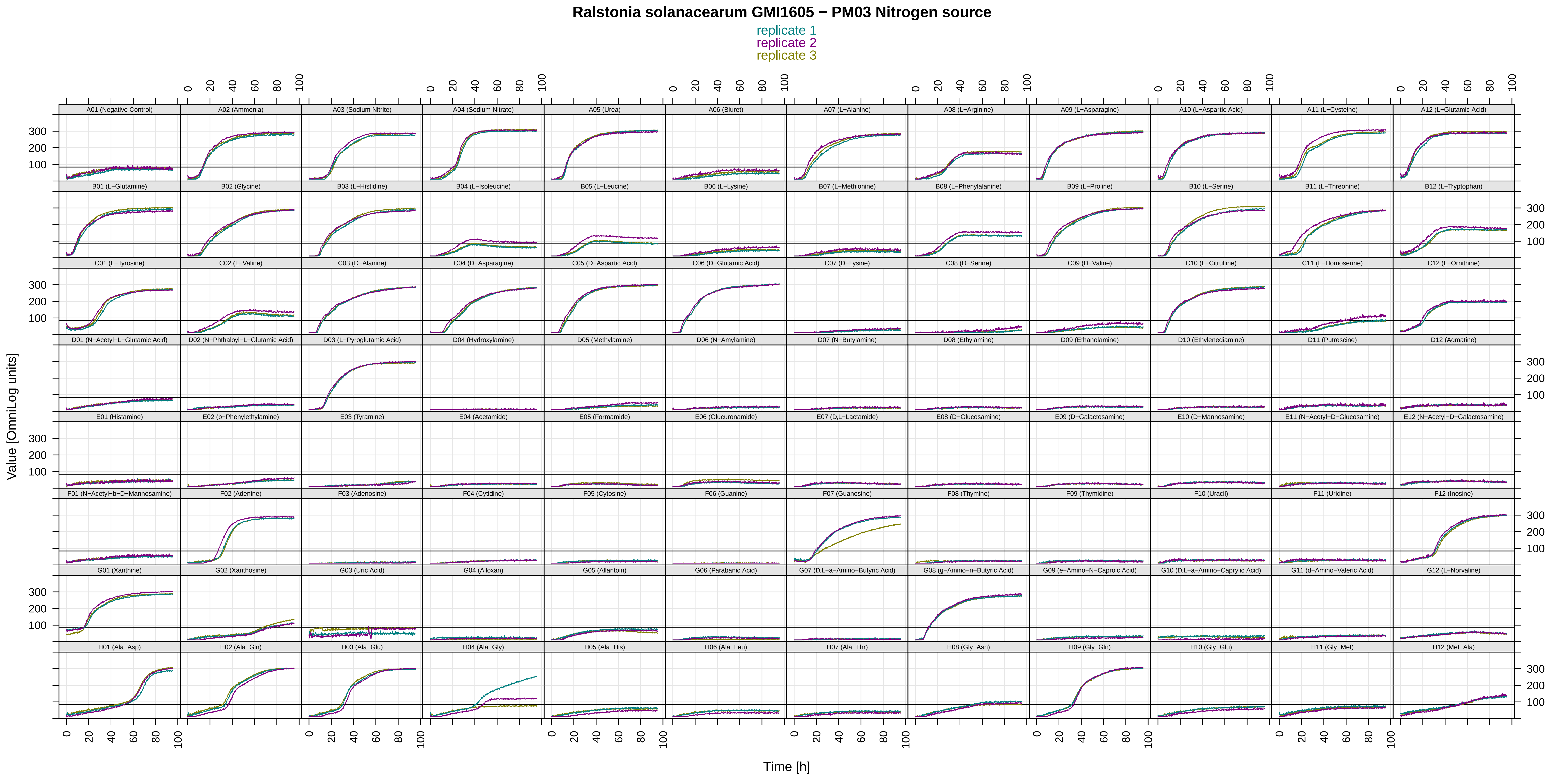
Ralstonia solanacearum GMI1605 − PM03 Nitrogen source
replicate 1
replicate 2
replicate 3
Value [OmniLog units]
Time [h]
A01 (Negative Control)
A02 (Ammonia)
A03 (Sodium Nitrite)
A04 (Sodium Nitrate)
A05 (Urea)
A06 (Biuret)
A07 (L−Alanine)
A08 (L−Arginine)
A09 (L−Asparagine)
A10 (L−Aspartic Acid)
A11 (L−Cysteine)
A12 (L−Glutamic Acid)
B01 (L−Glutamine)
B02 (Glycine)
B03 (L−Histidine)
B04 (L−Isoleucine)
B05 (L−Leucine)
B06 (L−Lysine)
B07 (L−Methionine)
B08 (L−Phenylalanine)
B09 (L−Proline)
B10 (L−Serine)
B11 (L−Threonine)
B12 (L−Tryptophan)
C01 (L−Tyrosine)
C02 (L−Valine)
C03 (D−Alanine)
C04 (D−Asparagine)
C05 (D−Aspartic Acid)
C06 (D−Glutamic Acid)
C07 (D−Lysine)
C08 (D−Serine)
C09 (D−Valine)
C10 (L−Citrulline)
C11 (L−Homoserine)
C12 (L−Ornithine)
D01 (N−Acetyl−L−Glutamic Acid)
D02 (N−Phthaloyl−L−Glutamic Acid)
D03 (L−Pyroglutamic Acid)
D04 (Hydroxylamine)
D05 (Methylamine)
D06 (N−Amylamine)
D07 (N−Butylamine)
D08 (Ethylamine)
D09 (Ethanolamine)
D10 (Ethylenediamine)
D11 (Putrescine)
D12 (Agmatine)
E01 (Histamine)
E02 (b−Phenylethylamine)
E03 (Tyramine)
E04 (Acetamide)
E05 (Formamide)
E06 (Glucuronamide)
E07 (D,L−Lactamide)
E08 (D−Glucosamine)
E09 (D−Galactosamine)
E10 (D−Mannosamine)
E11 (N−Acetyl−D−Glucosamine)
E12 (N−Acetyl−D−Galactosamine)
F01 (N−Acetyl−b−D−Mannosamine)
F02 (Adenine)
F03 (Adenosine)
F04 (Cytidine)
F05 (Cytosine)
F06 (Guanine)
F07 (Guanosine)
F08 (Thymine)
F09 (Thymidine)
F10 (Uracil)
F11 (Uridine)
F12 (Inosine)
G01 (Xanthine)
G02 (Xanthosine)
G03 (Uric Acid)
G04 (Alloxan)
G05 (Allantoin)
G06 (Parabanic Acid)
G07 (D,L−a−Amino−Butyric Acid)
G08 (g−Amino−n−Butyric Acid)
G09 (e−Amino−N−Caproic Acid)
G10 (D,L−a−Amino−Caprylic Acid)
G11 (d−Amino−Valeric Acid)
G12 (L−Norvaline)
H01 (Ala−Asp)
H02 (Ala−Gln)
H03 (Ala−Glu)
H04 (Ala−Gly)
H05 (Ala−His)
H06 (Ala−Leu)
H07 (Ala−Thr)
H08 (Gly−Asn)
H09 (Gly−Gln)
H10 (Gly−Glu)
H11 (Gly−Met)
H12 (Met−Ala)

Ralstonia solanacearum GMI1755 − PM01 Carbon source

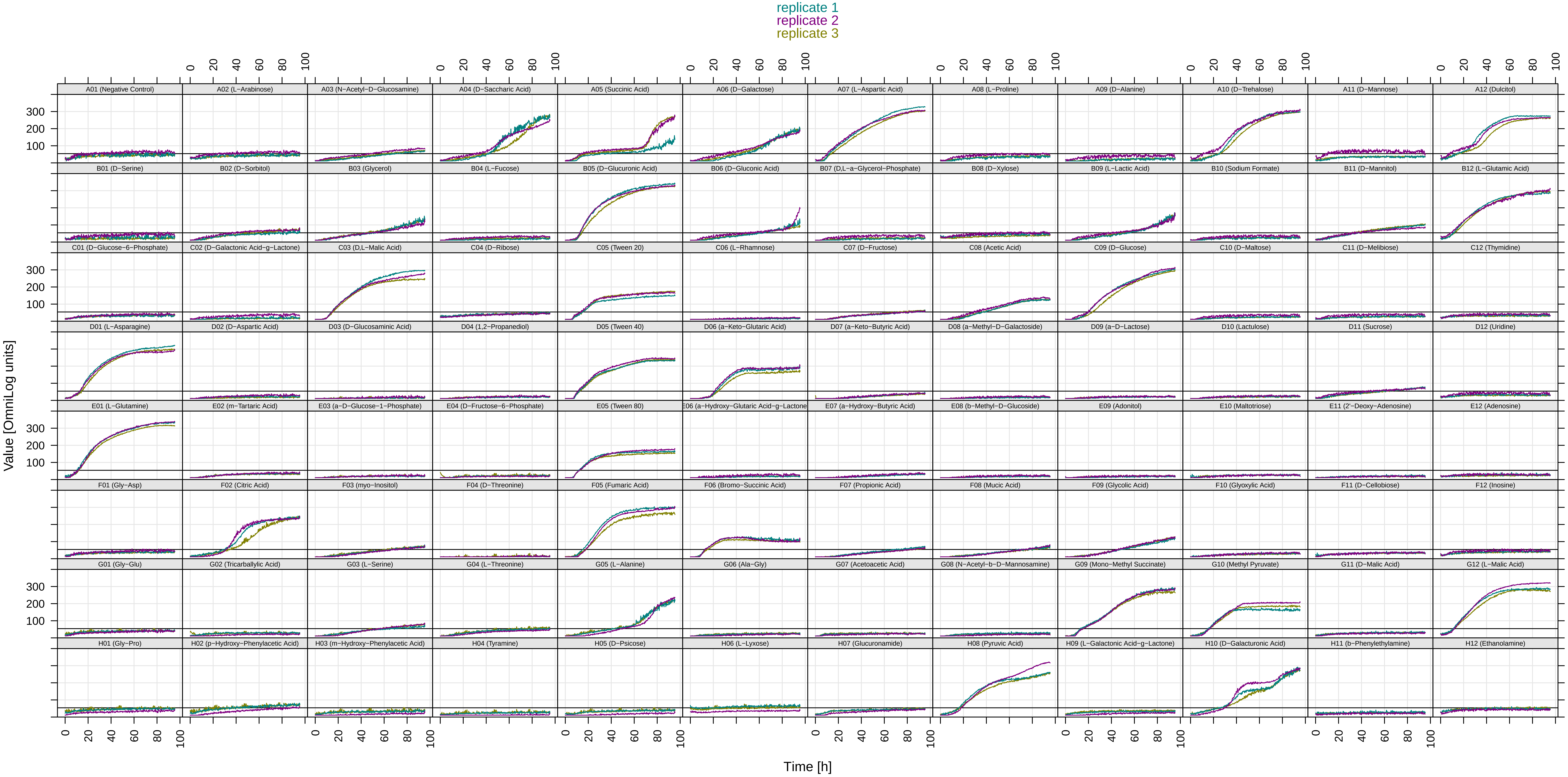

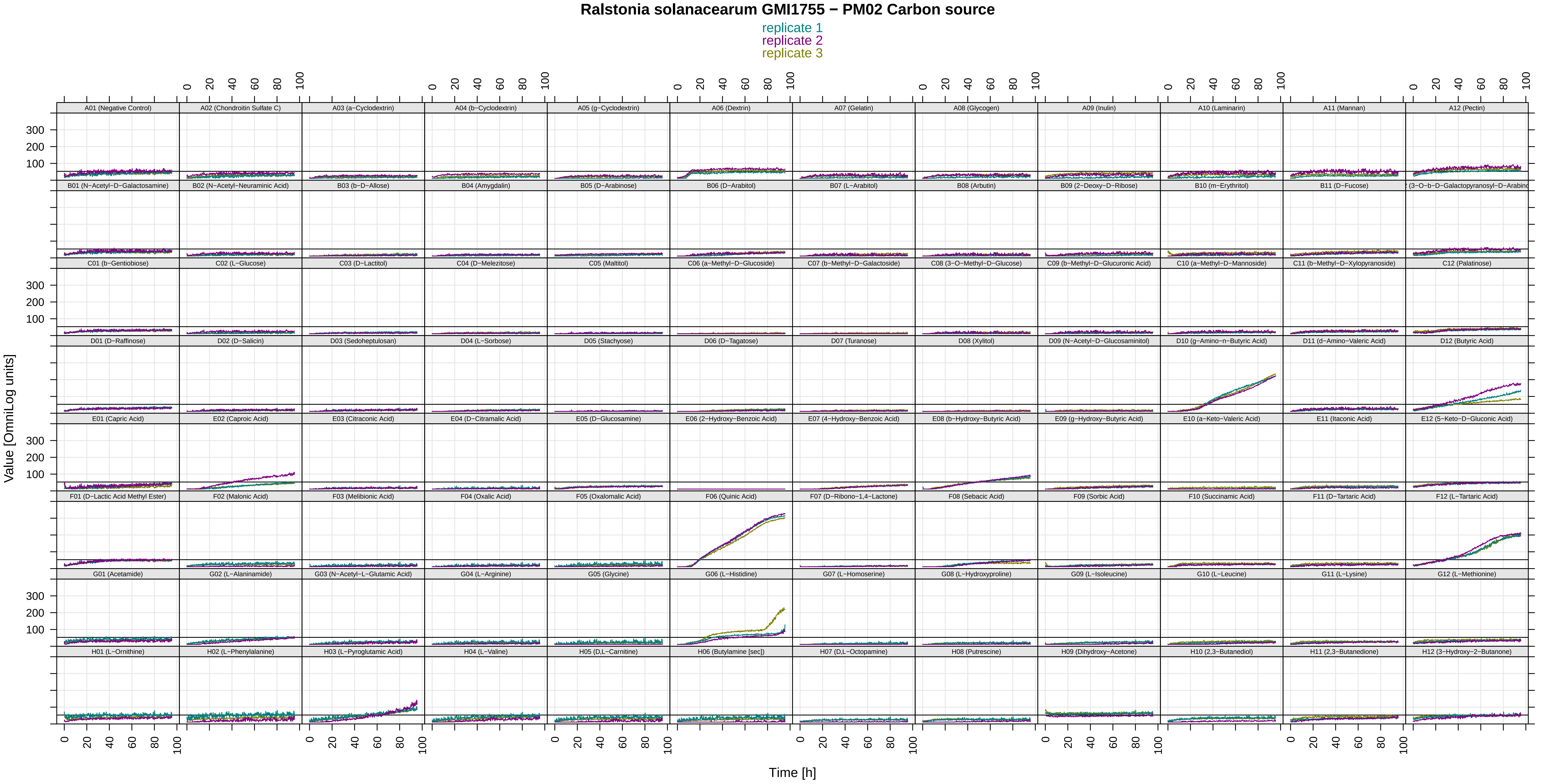
Ralstonia solanacearum GMI1755 − PM02 Carbon source
replicate 1
replicate 2
replicate 3
Value [OmniLog units]
Time [h]
0
20
40
60
80
100
100
200
300
A01 (Negative Control)
A02 (Chondroitin Sulfate C)
A03 (a−Cyclodextrin)
A04 (b−Cyclodextrin)
A05 (g−Cyclodextrin)
A06 (Dextrin)
A07 (Gelatin)
A08 (Glycogen)
A09 (Inulin)
A10 (Laminarin)
A11 (Mannan)
A12 (Pectin)
B01 (N−Acetyl−D−Galactosamine)
B02 (N−Acetyl−Neuraminic Acid)
B03 (b−D−Allose)
B04 (Amygdalin)
B05 (D−Arabinose)
B06 (D−Arabitol)
B07 (L−Arabitol)
B08 (Arbutin)
B09 (2−Deoxy−D−Ribose)
B10 (m−Erythritol)
B11 (D−Fucose)
(3−O−b−D−Galactopyranosyl−D−Arabino
C01 (b−Gentiobiose)
C02 (L−Glucose)
C03 (D−Lactitol)
C04 (D−Melezitose)
C05 (Maltitol)
C06 (a−Methyl−D−Glucoside)
C07 (b−Methyl−D−Galactoside)
C08 (3−O−Methyl−D−Glucose)
C09 (b−Methyl−D−Glucuronic Acid)
C10 (a−Methyl−D−Mannoside)
C11 (b−Methyl−D−Xylopyranoside)
C12 (Palatinose)
D01 (D−Raffinose)
D02 (D−Salicin)
D03 (Sedoheptulosan)
D04 (L−Sorbose)
D05 (Stachyose)
D06 (D−Tagatose)
D07 (Turanose)
D08 (Xylitol)
D09 (N−Acetyl−D−Glucosaminitol)
D10 (g−Amino−n−Butyric Acid)
D11 (d−Amino−Valeric Acid)
D12 (Butyric Acid)
E01 (Capric Acid)
E02 (Caproic Acid)
E03 (Citraconic Acid)
E04 (D−Citramalic Acid)
E05 (D−Glucosamine)
E06 (2−Hydroxy−Benzoic Acid)
E07 (4−Hydroxy−Benzoic Acid)
E08 (b−Hydroxy−Butyric Acid)
E09 (g−Hydroxy−Butyric Acid)
E10 (a−Keto−Valeric Acid)
E11 (Itaconic Acid)
E12 (5−Keto−D−Gluconic Acid)
F01 (D−Lactic Acid Methyl Ester)
F02 (Malonic Acid)
F03 (Melibionic Acid)
F04 (Oxalic Acid)
F05 (Oxalomalic Acid)
F06 (Quinic Acid)
F07 (D−Ribono−1,4−Lactone)
F08 (Sebacic Acid)
F09 (Sorbic Acid)
F10 (Succinamic Acid)
F11 (D−Tartaric Acid)
F12 (L−Tartaric Acid)
G01 (Acetamide)
G02 (L−Alaninamide)
G03 (N−Acetyl−L−Glutamic Acid)
G04 (L−Arginine)
G05 (Glycine)
G06 (L−Histidine)
G07 (L−Homoserine)
G08 (L−Hydroxyproline)
G09 (L−Isoleucine)
G10 (L−Leucine)
G11 (L−Lysine)
G12 (L−Methionine)
H01 (L−Ornithine)
H02 (L−Phenylalanine)
H03 (L−Pyroglutamic Acid)
H04 (L−Valine)
H05 (D,L−Carnitine)
H06 (Butylamine [sec])
H07 (D,L−Octopamine)
H08 (Putrescine)
H09 (Dihydroxy−Acetone)
H10 (2,3−Butanediol)
H11 (2,3−Butanedione)
H12 (3−Hydroxy−2−Butanone)

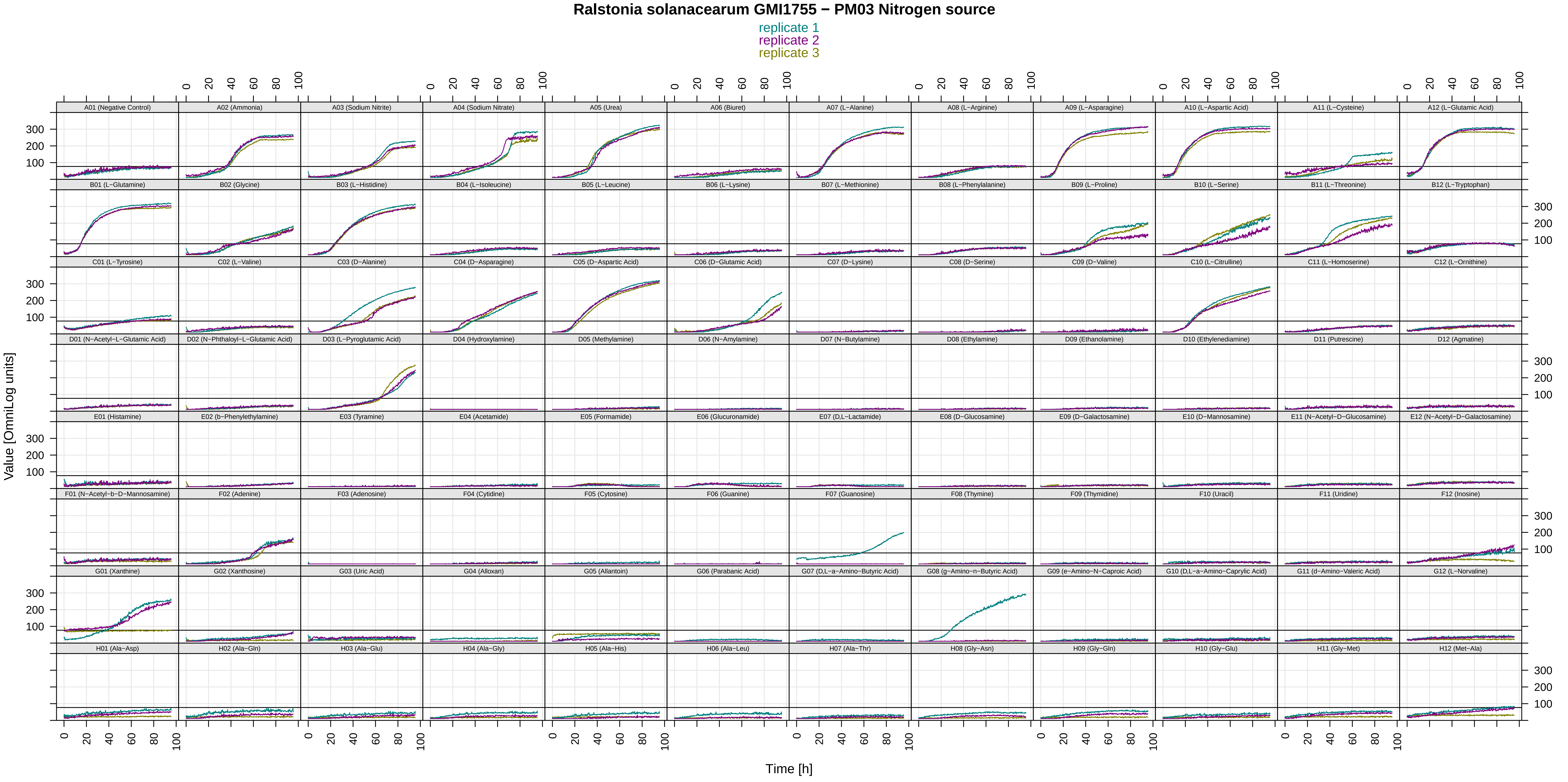
Ralstonia solanacearum GMI1755 − PM03 Nitrogen source
replicate 1
replicate 2
replicate 3
A01 (Negative Control)
A02 (Ammonia)
A03 (Sodium Nitrite)
A04 (Sodium Nitrate)
A05 (Urea)
A06 (Biuret)
A07 (L−Alanine)
A08 (L−Arginine)
A09 (L−Asparagine)
A10 (L−Aspartic Acid)
A11 (L−Cysteine)
A12 (L−Glutamic Acid)
B01 (L−Glutamine)
B02 (Glycine)
B03 (L−Histidine)
B04 (L−Isoleucine)
B05 (L−Leucine)
B06 (L−Lysine)
B07 (L−Methionine)
B08 (L−Phenylalanine)
B09 (L−Proline)
B10 (L−Serine)
B11 (L−Threonine)
B12 (L−Tryptophan)
C01 (L−Tyrosine)
C02 (L−Valine)
C03 (D−Alanine)
C04 (D−Asparagine)
C05 (D−Aspartic Acid)
C06 (D−Glutamic Acid)
C07 (D−Lysine)
C08 (D−Serine)
C09 (D−Valine)
C10 (L−Citrulline)
C11 (L−Homoserine)
C12 (L−Ornithine)
D01 (N−Acetyl−L−Glutamic Acid)
D02 (N−Phthaloyl−L−Glutamic Acid)
D03 (L−Pyroglutamic Acid)
D04 (Hydroxylamine)
D05 (Methylamine)
D06 (N−Amylamine)
D07 (N−Butylamine)
D08 (Ethylamine)
D09 (Ethanolamine)
D10 (Ethylenediamine)
D11 (Putrescine)
D12 (Agmatine)
E01 (Histamine)
E02 (b−Phenylethylamine)
E03 (Tyramine)
E04 (Acetamide)
E05 (Formamide)
E06 (Glucuronamide)
E07 (D,L−Lactamide)
E08 (D−Glucosamine)
E09 (D−Galactosamine)
E10 (D−Mannosamine)
E11 (N−Acetyl−D−Glucosamine)
E12 (N−Acetyl−D−Galactosamine)
F01 (N−Acetyl−b−D−Mannosamine)
F02 (Adenine)
F03 (Adenosine)
F04 (Cytidine)
F05 (Cytosine)
F06 (Guanine)
F07 (Guanosine)
F08 (Thymine)
F09 (Thymidine)
F10 (Uracil)
F11 (Uridine)
F12 (Inosine)
G01 (Xanthine)
G02 (Xanthosine)
G03 (Uric Acid)
G04 (Alloxan)
G05 (Allantoin)
G06 (Parabanic Acid)
G07 (D,L−a−Amino−Butyric Acid)
G08 (g−Amino−n−Butyric Acid)
G09 (e−Amino−N−Caproic Acid)
G10 (D,L−a−Amino−Caprylic Acid)
G11 (d−Amino−Valeric Acid)
G12 (L−Norvaline)
H01 (Ala−Asp)
H02 (Ala−Gln)
H03 (Ala−Glu)
H04 (Ala−Gly)
H05 (Ala−His)
H06 (Ala−Leu)
H07 (Ala−Thr)
H08 (Gly−Asn)
H09 (Gly−Gln)
H10 (Gly−Glu)
H11 (Gly−Met)
H12 (Met−Ala)
Value [OmniLog units]
Time [h]
0
20
40
60
80
100
100
200
300
