# Supplementary material for: A Resource Allocation Trade-Off between Virulence and Proliferation Drives Metabolic Versatility in the Plant Pathogen Ralstonia solanacearum
Source: PLoS Pathog. 2016 Oct 12;12(10):e1005939. doi: 10.1371/journal.ppat.1005939 (PMC5061431; doi:10.1371/journal.ppat.1005939)
Supplement: S1 Material — Supplementary material containing details on the reconstruction pipelines, and the various in silico analyses. Detail of the algorithms used for in silico analyses and the corresponding scripts are available and can be freely downloaded at the following location: http://lipm-bioinfo.toulouse.inra.fr/systemsbiology/models/rsolanacearum. (PDF) [file ppat.1005939.s008.pdf]

Supplementary Material S1 for

A resource allocation Trade-off between virulence and proliferation drives metabolic versatility in the plant pathogen *Ralstonia solanacearum*

Rémi Peyraud, Ludovic Cottret, Lucas Marmiesse, Jérôme Gouzy, Stéphane Genin

Contents

I. Metabolic network reconstruction____2

A. Overview

B. Automatic reconstruction of several draft metabolic models from curated metabolic models of other species based on orthology

C. Semi-automatic standardisation of the draft metabolic models

D. Semi-automatic reconciliation of the standardised draft metabolic models in one high-quality draft metabolic model

E. Determination of the biomass function.

F. Validation

1) Metabolic Flux Analysis

2) Comparison between *in silico* phenotype predictions and phenotype microarray plates

II. Bibliography____13

I. Metabolic network reconstruction

A. Overview

The reconstruction of the metabolic model of *R. solanacearum* has been performed as described in the Thiele and Palsson's protocol (1) excepting for the generation of the draft reconstruction (Supplementary Material 1 Supplementary Material 1 Figure 1). Indeed, we developed automatic and semi automatic tools to build a first high quality draft metabolic model that required less corrections and refinements than a draft metabolic model built with classical methods. Each bioinformatics tool described below responds to the same objective: accelerating speed of the draft generation without sacrificing high accuracy.

The first step of the high quality draft reconstruction of the metabolic model of *R.solanacearum* has been to produce draft metabolic models built from four metabolic models from other bacteria (see Section I. B).
Since these metabolic models use different ontologies, the second step has been to standardize the identifiers of the four draft metabolic models (see Section I. C).
At last, the four standardised draft metabolic models were merged in one high quality draft metabolic model (see Section I. D).

All the tools we developed have been designed in a generic way that allows using them for other future metabolic reconstructions.

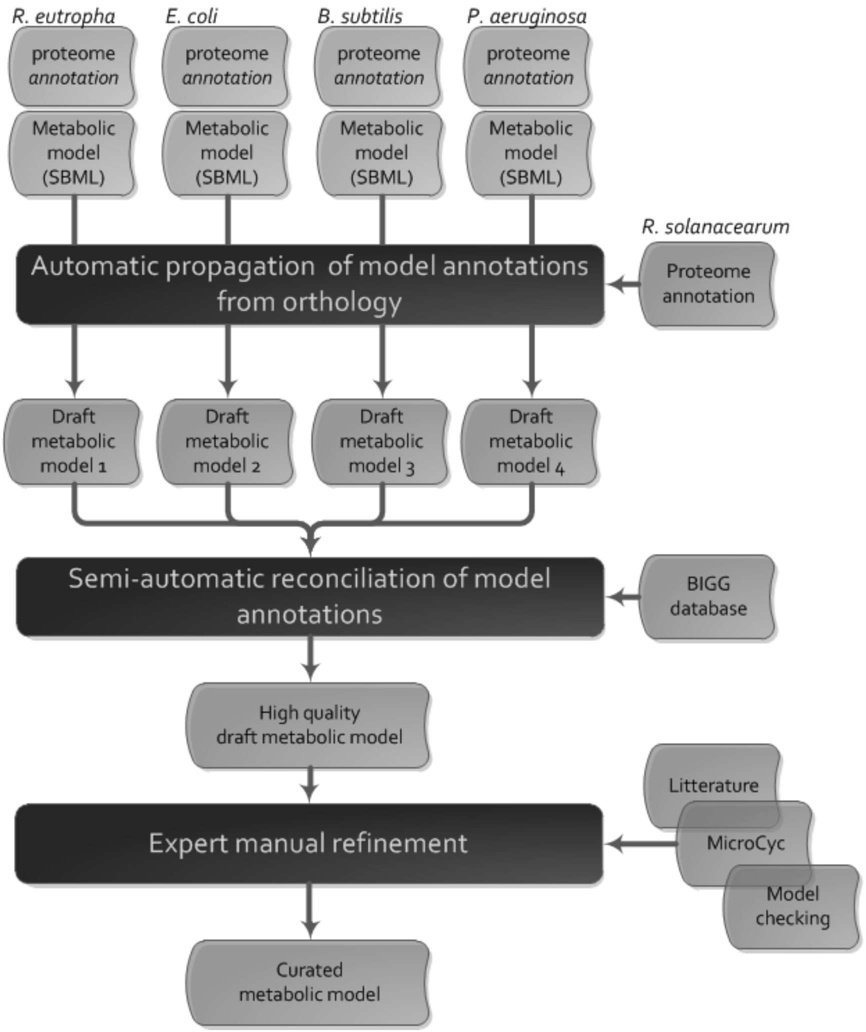

Supplementary Material 1 Figure 1. Metabolic network reconstruction of R. solanacearum

B. Automatic reconstruction of several draft metabolic models from curated metabolic models of other species based on orthology

Draft metabolic models have been built from these four published metabolic models:

- *Ralstonia eutropha* (RehMBEL1391 (2))
- *Bacillus subtilis* (Bs_iYO844 (3))
- *Pseudomonas aeruginosa* (iMO1086 (4))
- *Escherichia coli* (iJO1366 (5))

P. aeruginosa, *E. coli*, and *R. eutropha* were selected because of their phylogenetic proximity with *R. solanacearum*. Furthermore, *P. aeruginosa* was also selected because of its pathogen lifestyle. The model of *B. subtilis* was used because of the high quality of its reconstruction.

The Systems Biology Markup Language (SBML) is for years the standard file format to exchange metabolic models (6). We naturally used this format for all our reconstruction and analysis steps. Three metabolic

models (*B. subtilis*, *P. aeruginosa*, and *E. coli*) were published in this format and so easily collected while the model of *R. eutropha* had to be converted from pdf to SBML since pdf was the only format supplied by the authors for their model.

We downloaded proteins sequences of the entire genome of the sources organisms from NCBI, plus the one of *R. solanacearum* strain GMI1000, later called the “target organism”, from the official genome web portal[1] (7).

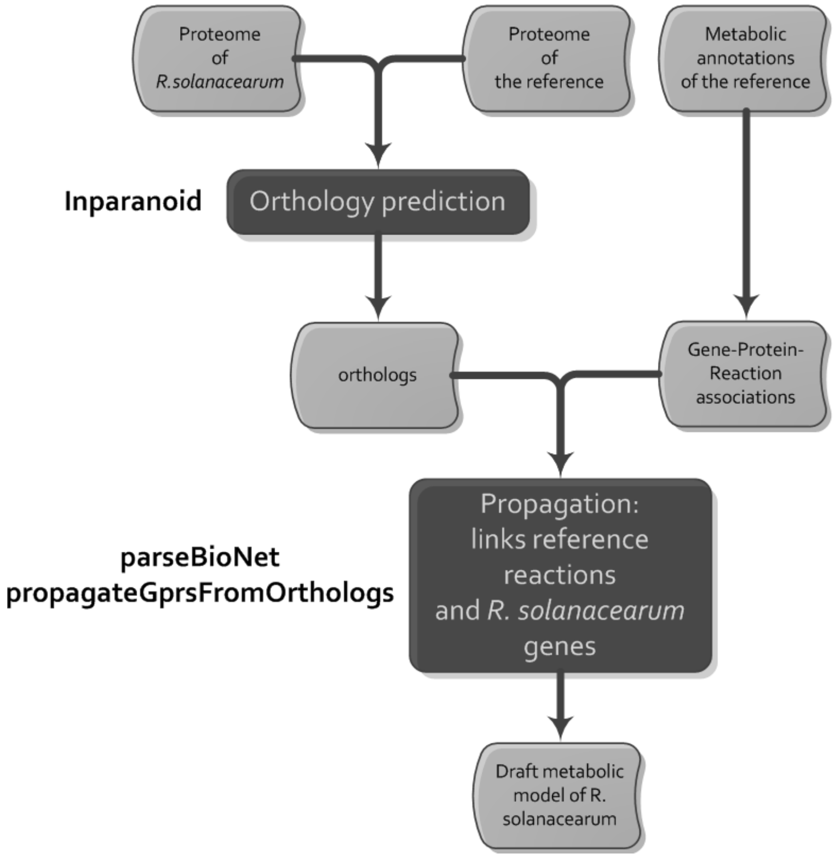

Supplementary Material 1 Figure 2. Propagation by orthology of the gene reaction association from a reference model to a draft model (inspired by the Autograph method (8))

We used the Autograph method (8) to automatically transfer by orthology the gene reaction associations of the four reference models in four draft models (Supplementary Material 1 Figure 2). The orthology prediction was made by using a tuned version of Inparanoid (9). Inparanoid found orthologs between the proteome of *R. solanacearum* and each of the four reference proteomes. The Blast results obtained in Inparanoid were filtered by only selecting hits for which the identity exceeds 30 % and the coverage exceeds 50 %. The BLOSUM45 matrix (reasonable for prokaryotes) was used for the Inparanoid bootstrapping step.

[1] **http://sequence.toulouse.inra.fr/R.solanacearum**

For the propagation step, we developed a function called PropagateGprsFromOrthologs in the parseBioNet package (JAVA package dedicated to metabolic networks that we develop for some years). PropagateGprsFromOrthologs takes as input the SBML reference file and a tabulated file containing for each protein of *R. solanacearum* the ortholog in the reference species, and returns a SBML file. Reactions in the reference model are included by PropagateGprsFromOrthologs in the target model if at least one reference gene has an ortholog in *R. solanacearum*. In the gene-protein-reaction links of the kept reactions, the identifiers of the genes are replaced by the identifiers of the corresponding orthologs in *R. solanacearum* or by the mention “no_ortholog” when the gene does not have any identified ortholog. Reactions without associated genes are propagated.

Results of the automatic propagation step are displayed in Supplementary Material 1 Figure 3. The proportion of reactions propagated from each reference model is quite high (> 60%). We will see in the next section that the manual curation performed after merging highly reduces this number.

Ortholog tables and SBML files produced by this step are available for downloading at the section Data of the web page http://lipm-bioinfo.toulouse.inra.fr/systemsbiology/models/rsolanacearum.

Our version of Inparanoid and the parseBioNet package are available for downloading at the section Tools of the web http://lipm-bioinfo.toulouse.inra.fr/systemsbiology/models/rsolanacearum.

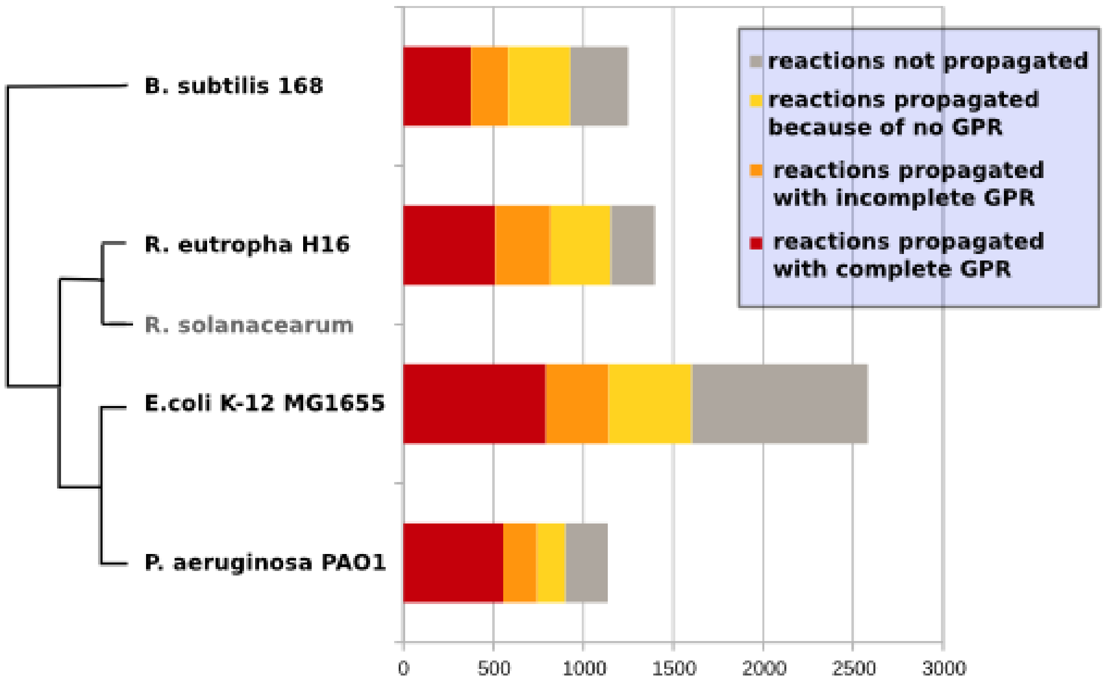

Supplementary Material 1 Figure 3. Propagation results. Number and type of reactions propagated from

each reference model. The tree at the left represents the taxonomy of the reference species and R. solanacearum. An interactive visualization of this plot is available at http://lipm-bioinfo.toulouse.inra.fr/systemsbiology/models/rsolanacearum/reconstruction/propagation

C. Semi-automatic standardisation of the draft metabolic models

Since the reference models have been made by different teams, they use different ontologies for their reaction and metabolite identifiers. For instance, the identifier of the adenosine triphosphate is *M_atp_c* in the models of *B.subtilis*, *E. coli* and *R. eutropha* while it's C0002 in the model of *P. aeruginosa*. So, before merging the four draft models, we needed to standardize the identifiers of their metabolites and reactions. Automatic reconciliation tools have been recently described *(10)*. However, they have some drawbacks. First, they often rely on information (Inchi, SMILES, cross-references) found in public metabolic databases to reconcile reaction and metabolite identifiers. Secondly, the process is always fully automatic and does not offer ways to complete or correct the produced dictionary. These two drawbacks are explained by the fact that these methods have been first essentially designed to quickly reconcile complete metabolic databases, such as KEGG *(11)*, MetaCyc *(12)* or BIGG. *(13)*.

For our topic, we wanted to quickly and without ambiguities reconcile the identifiers of the four draft metabolic models produced by the propagation step. For this, we developed a web-designed semi automatic tool called SAMIR (**S**emi **A**utomatic **M**etabolic **I**dentifier **R**econciliation). SAMIR is generic since the standard identifiers (as well as the identifiers to standardize) are provided by a SBML file (the standard format for metabolic networks *(6)*) that can be built from public or home-made metabolic database. In the same way than MNXref *(10)*, the process of the reconciliation of metabolites use the reaction context. If two reactions are identified as identical in the two SBML files, then mapping between the metabolites involved in these reactions is proposed. In the same way, if two reactions involve metabolites identified as identical, they will be proposed for the reconciliation. The main difference between MNXRef and SAMIR is that the former tries to validate the potential mappings and iterates until no new mappings can be obtained while the latter stops after each iteration and let the user to decide which mapping is the best. The second alternative is certainly more time consuming but has the great advantage to obtain an on-the-fly curated reconciliation at the end. A system of score has been designed to help the user to decide between several mappings and the potential mappings are sorted by their score in the interface (**Erreur ! Source du renvoi introuvable.**.*a*). For both metabolites and reactions, the score includes a measure of the name similarity. For reactions, the score includes also the percentage of metabolites identified as identical and a bonus if the two EC numbers are identical. For metabolites, the score includes bonus if the chemical formulae (potentially provided with the SBML file) are the same or if the pair to check is the last one not mapped in a pair of identical reactions. Matching entities and ranking of probable matches are listed in the

web interface allowing annotators to curate matches. The web interface allows to declare some *a priori* on the identical identifiers (provided for instance by the methods cited above), on the identifiers that are definitely different (and that won't be proposed for the reconciliation) and on the identifiers that are unique to the network to standardize and that can be added at the end to the reference SBML file. To our knowledge, SAMIR is moreover the first reconciliation method which takes into account compartments in the models by declaring which compartments are identical in the two SBML files. After each iteration, a pie chart indicates to the user the progression of the reconciliation (**Erreur ! Source du renvoi introuvable.**.b) and displays the links to the standardized SBML file, the produced dictionary and the reference SBML file completed with the reactions unique to the SBML file to standardize. Since SAMIR is generic and publicly available in a Beta version[2], it can be used to reconcile other metabolic reconstructions.

[2] http://metexplore.toulouse.inra.fr/metexploreJoomla/metexplore/metannot/toolbox/standardize.php

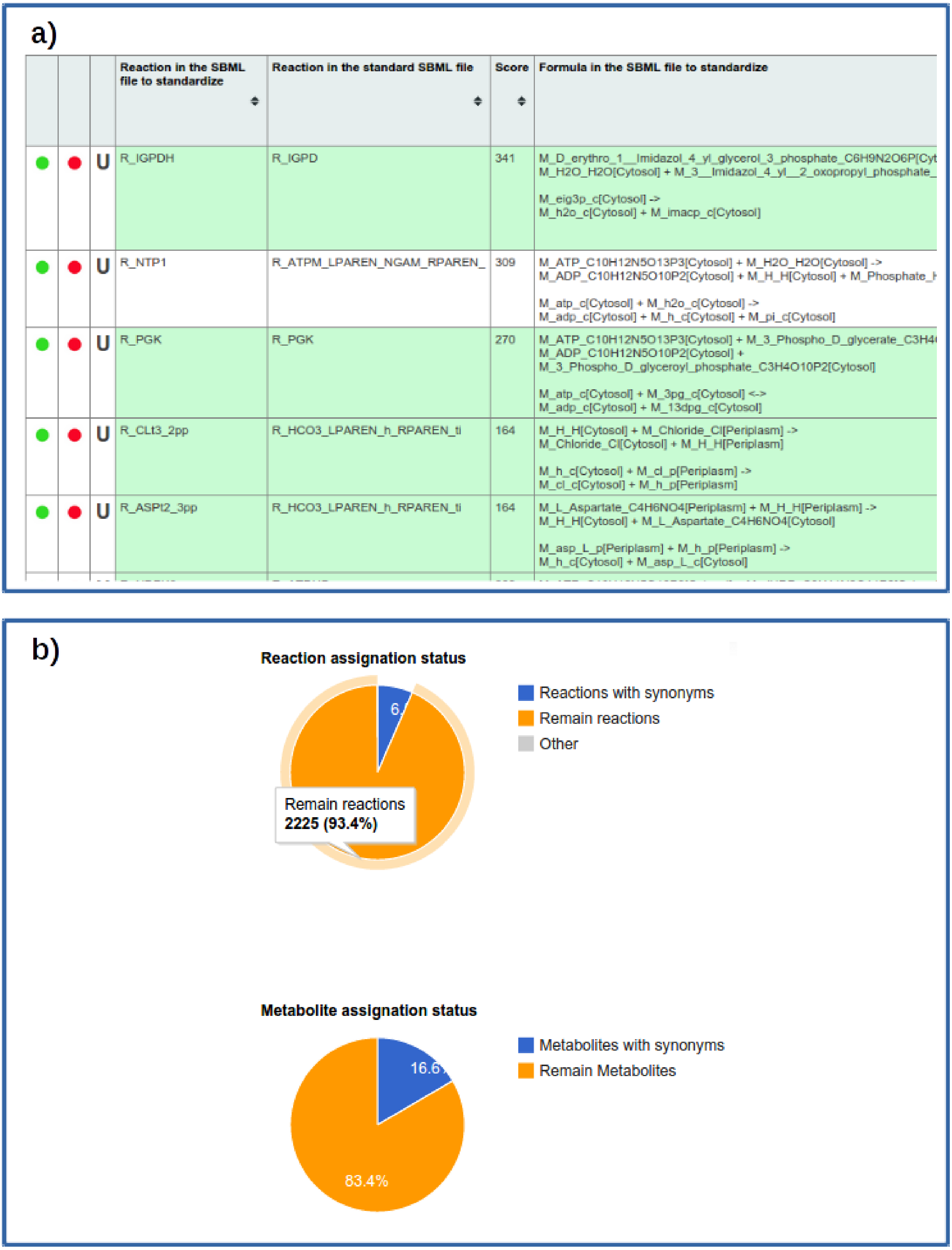

Supplementary Material 1 Figure 4. SAMIR (Semi Automatic Metabolic Identifier Reconciliation) screenshots. ***a)*** *Interface to identify identical reactions (green button), distinct reactions (red button) and reactions unique to the network to standardize (U button). Reactions and metabolites are sorted by their scores.* ***b)****Pie charts indicating the progress of the reconciliation after each iteration.*

We launched SAMIR to build four standardized metabolic models from the four draft models built by propagation. To be able to compare our model with most of the metabolic models, we chose the BIGG database for which the identifiers have been used to build most of the existing metabolic models. We can see in Supplementary Material 1 Figure 5 that only 40 propagated reactions are found in every reference model. Also, a lot of the reactions (515) propagated in the final model exclusively come from *E. coli*. This can easily be explained by the completeness of this model which is not found in the other ones. Numerous reactions have been manually removed from the propagation results (e.g. 671 reactions from *E.coli*). Indeed, orthology evidence is often not sufficient to add a reaction in the model. The metabolic context, deduced by the other reactions and by the literature, thus made reactions dismissed. At last, 853 reactions which don't come from the propagation results have been manually added to the final model (see next section). This highlights the importance of manual curation after a propagation step.

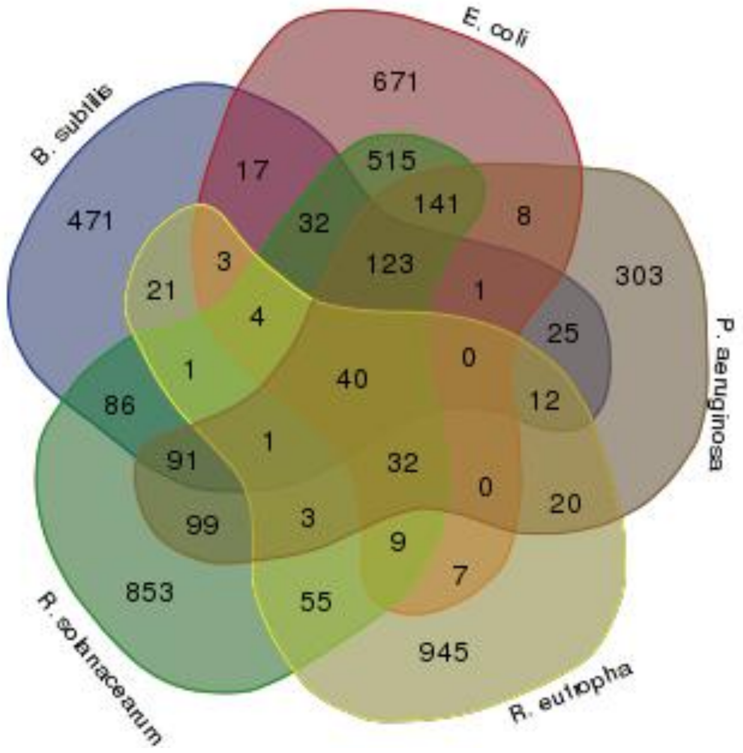

Supplementary Material 1 Figure 5. Venn diagram representing the intersection between each model built by propagation and the final model (in green). Built thanks to the web interface: http://bioinformatics.psb.ugent.be/webtools/Venn/.

An interactive visualisation is available here:

http://lipm-bioinfo.toulouse.inra.fr/systemsbiology/models/rsolanacearum/reconstruction/merging

D. Semi-automatic reconciliation of the standardised draft metabolic models in one high-quality draft metabolic model

The resulting four propagated networks into the target organism are merged following an expert evaluation of the best reactions candidates propagated from the sources considering the completeness of the GPRs, ranking of blast results, and quality of the annotation in the source GEMs. We developed a parseBioNet application called CompareSbmls which returns information about reactions shared between models, gene protein reaction links shared between reactions to accelerate this step.

E. Determination of the biomass function

List of the biomass composition used to build the biomass function of *R. solanacearum* GMI1000 can be found in the Supplementary table 02. We determined the cell dry weigh (CDW) to optical density (OD) correlation factor experimentally (see Material and Methods) and found a factor of 0.414 ± 0.042 $g_{(CDW)}.OD_{(600nm)}^{-1}.l^{-1}$. The protein content of the cell was found to be 52.9% ± 4.7 in $g_{(protein)}.g_{(CDW)}^{-1}$.

F. Validation

1. Metabolic flux analysis

Metabolic fluxes of substrates consumption and production of metabolites and macromolecules was calculated from the kinetic of their concentration in the growth medium determined by NMR (see Material and Methods). The equation used to fit the consumption rate (Qs) to the concentration data is the following:

$$[C]_{(t)} = C_{(0)} + Qs/\mu*X_{(0)}*(1-\exp(\mu*t))$$

Where [C] is the concentration of the compound in the medium, X is the initial concentration of the biomass in g, μ is the growth rate in h^{-1}, t is the time in hour.

2. Comparison between *in silico* phenotype predictions and phenotype microarray plates

Phenotypic microarray results were reduced to a Boolean vector indicating for each Biolog condition the presence or the absence of growth.
Biolog conditions were converted in a Boolean matrix indicating the presence or the absence of a substrate in the medium for each condition. Then, thanks to the function BECO of FlexFlux, Boolean values were automatically converted into flux constraints and optimal growth rate was computed by Flux Balance

Analysis (see Section IIIA) for each of them. Finally, *in silico* data was also transformed into a Boolean vector and compared to the experimentally defined one (Supplementary Material 1 Figure 6). Complete results can be found in Supplementary Table 4 and in a web interactive table at http://lipm-bioinfo.toulouse.inra.fr/systemsbiology/models/rsolanacearum/biolog.html.

The number of true positive predictions (TP) corresponds here to the number of conditions for which both experimental and *in silico* methods found a positive growth. The number of positive predictions (P) corresponds to the number of conditions for which the *in silico* method found a positive growth. The number of false negative predictions (FN) corresponds to the number of conditions for which the *in silico* method found a null growth while the experimental method found a positive growth.

Sensitivity and specificity have been computed to formula described in Material and Methods.

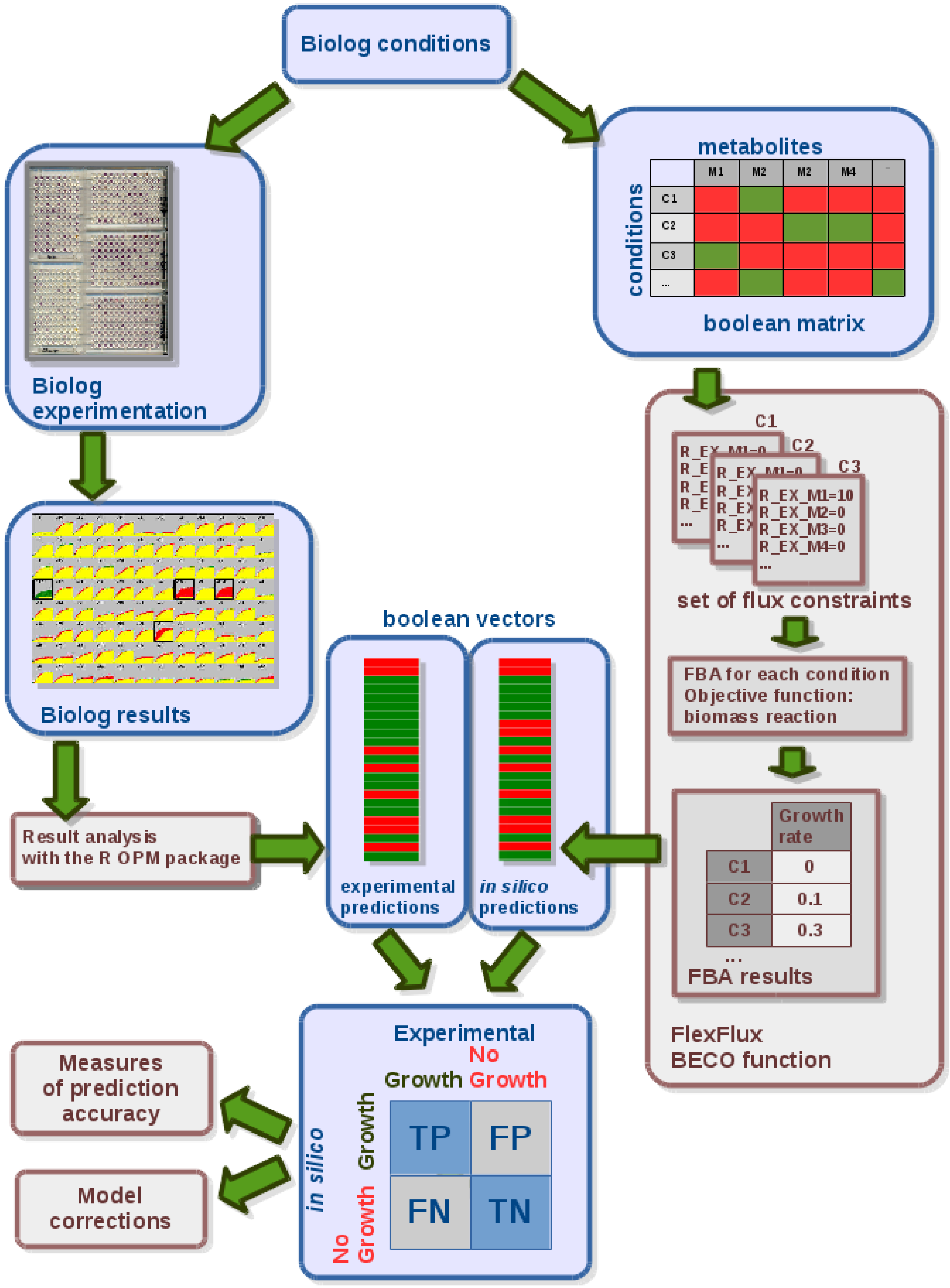

Supplementary Material 1 Figure 6. Metabolic network validation by comparing Biolog results and in silico growth predictions

IV. Bibliography

1. Thiele I, Palsson BØ. A protocol for generating a high-quality genome-scale metabolic reconstruction. Nat Protoc. 2010 Jan;5(1):93–121.

2. Park JM, Kim TY, Lee SY. Genome-scale reconstruction and in silico analysis of the Ralstonia eutropha H16 for polyhydroxyalkanoate synthesis, lithoautotrophic growth, and 2-methyl citric acid production. BMC Syst Biol. 2011 Jan;5:101.

3. Oh Y-K, Palsson BO, Park SM, Schilling CH, Mahadevan R. Genome-scale reconstruction of metabolic network in Bacillus subtilis based on high-throughput phenotyping and gene essentiality data. J Biol Chem. 2007 Sep 28;282(39):28791–9.

4. Oberhardt MA, Puchałka J, Martins dos Santos VAP, Papin JA. Reconciliation of genome-scale metabolic reconstructions for comparative systems analysis. Bourne PE, editor. PLoS Comput Biol. 2011 Mar;7(3):e1001116.

5. Orth JD, Conrad TM, Na J, Lerman JA, Nam H, Feist AM, et al. A comprehensive genome-scale reconstruction of Escherichia coli metabolism—2011. Mol Syst Biol. 2011 Oct 11;7.

6. Hucka M, Finney A, Sauro HM, Bolouri H, Doyle JC, Kitano H, et al. The systems biology markup language (SBML): a medium for representation and exchange of biochemical network models. Bioinformatics. 2003 Mar;19(4):524–31.

7. Salanoubat M, Genin S, Artiguenave F, Gouzy J, Mangenot S, Arlat M, et al. Genome sequence of the plant pathogen Ralstonia solanacearum. Nature. 2002 Jan 31;415(6871):497–502.

8. Notebaart RA, van Enckevort FHJ, Francke C, Siezen RJ, Teusink B. Accelerating the reconstruction of genome-scale metabolic networks. BMC Bioinformatics. 2006 Jan;7(1):296.

9. Remm M, Storm CE, Sonnhammer EL. Automatic clustering of orthologs and in-paralogs from pairwise species comparisons. J Mol Biol. 2001 Dec 14;314(5):1041–52.

10. Bernard T, Bridge A, Morgat A, Moretti S, Xenarios I, Pagni M. Reconciliation of metabolites and biochemical reactions for metabolic networks. Brief Bioinform. 2012 Dec 3;bbs058 – .

11. Kanehisa M, Goto S. KEGG: kyoto encyclopedia of genes and genomes. Nucleic Acids Res. 2000 Jan 1;28(1):27–30.

12. Caspi R, Altman T, Billington R, Dreher K, Foerster H, Fulcher CA, et al. The MetaCyc database of metabolic pathways and enzymes and the BioCyc collection of Pathway/Genome Databases. Nucleic Acids Res. 2014 Jan 1;42(Database issue):D459–71.

13. Schellenberger J, Park JO, Conrad TM, Palsson BØ. BiGG: a Biochemical Genetic and Genomic knowledgebase of large scale metabolic reconstructions. BMC Bioinformatics. 2010 Jan;11(1):213.
