# Supplementary material for: A Resource Allocation Trade-Off between Virulence and Proliferation Drives Metabolic Versatility in the Plant Pathogen Ralstonia solanacearum
Source: PLoS Pathog. 2016 Oct 12;12(10):e1005939. doi: 10.1371/journal.ppat.1005939 (PMC5061431; doi:10.1371/journal.ppat.1005939)
Supplement: S3 Material — (PDF) [file ppat.1005939.s010.pdf]

Supplementary Material S3 for

Trade-off between virulence and proliferation drives metabolic versatility in a the plant pathogen *Ralstonia solanacearum*

Rémi Peyraud, Ludovic Cottret, Lucas Marmiesse, Jérôme Gouzy, Stéphane Genin

Definition of substrate usage capacity

The substrate usage capacity of a biological system can be defined as the production of an output – such as proliferation or virulence factor production - that the cell can produce from a given substrate upon a period of time. Hence, the substrate usage capacity depends on i) the rate of the substrate collected in the environment, i.e. substrate uptake rate, and ii) the efficiency of the conversion of this substrate into the specific output, i.e the yield through the metabolic network. The yield depends on the topology of the metabolic network mobilized whereas the rate of the transporters and the metabolic pathways mobilized depends on the investment of the cell into the transporters and the enzymes catalyzing the reactions. In fact, the substrate usage capacity corresponds to flux of production of a studied output using a limiting substrate. Thus, it can be measured experimentally by metabolic flux analysis or its maximal value can be calculated by Flux balanced Analysis if the substrate uptake rate is known.

Substrate usage capacity for competing traits (e.g. proliferation vs virulence)

Rerouting part of the metabolic fluxes from the limiting substrate toward a second competing trait (like virulence) will decrease the maximal output of a first trait (proliferation). Several mechanisms that could work in conjunction can be responsible for this decrease. First, for a same amount of substrate assimilated, the yield of the first trait will decrease from a proportion corresponding to the amount of resources rerouted toward the second trait.

This corresponds to a decrease of the yield toward the first trait. In fact, considering the complexity of metabolic networks, the trade-off in resource allocation may not be always linear since some pathways can harbor a certain degree of complementarity. For instance if the optimal flux distribution of the first trait generates an over production of NADPH and the second trait is NADPH limited, both traits can harbor a certain degree of complementarity. The second mechanism inherent to biological system leading to a decrease the maximal output of the first trait can emerge from the enzymes crowding inside the cell. Indeed, there is a maximal set of enzymes that can be expressed in cells having a limited volume and shape. Thus, the expression of the enzymes set devoted to the second trait might lead to decrease the concentration of enzymes devoted to the first trait or even the transporters required for the substrate acquisition. All together, they will decrease the rate of the reactions devoted to the first trait.
